# Effect of time-weighted average 25(OH)D on the occurrence of major adverse kidney events in IgA nephropathy—from a 10-year population-based cohort study

**DOI:** 10.3389/fnut.2025.1576488

**Published:** 2025-08-01

**Authors:** Fang Lu, Chang Liu, Dandan Song, Li Qian, Jingfeng Zhu, Jingjing Wu, Chengning Zhang, Zhimin Huang, Ming Zeng, Bin Sun, Bo Zhang, Suyan Duan, Yanggang Yuan, Changying Xing, Huijuan Mao

**Affiliations:** Department of Nephrology, The First Affiliated Hospital with Nanjing Medical University, Nanjing Medical University, Nanjing, China

**Keywords:** IgAN, 25(OH)D, major adverse kidney events, scRNA-seq analysis, molecular docking analysis

## Abstract

**Background:**

Vitamin D (VD) deficiency has been found to be common and associated with a higher risk of adverse outcomes in chronic kidney disease (CKD), according to certain studies. However, whether it is associated with the progression of IgA nephropathy (IgAN) and the efficacy of supplementation remains a topic of debate.

**Methods:**

A total of 866 patients with IgAN were included. Identification of the baseline and time-weighted average (TWA) serum 25-hydroxyvitamin D (25(OH)D) levels associated with the major adverse kidney events (MAKE) was performed using Kaplan–Meier survival analysis, receiver operating characteristic (ROC) curves, and multivariate logistic regression analysis. Furthermore, the dataset was divided into derivation and validation cohorts using a 6:4 ratio. Internal validation was performed to assess the added value of TWA 25(OH)D levels to clinical variables using ROC curves, decision curve analysis, and Net Reclassification Improvement (NRI). An integrative analysis combining genomic, single-cell RNA sequencing (scRNA-seq), and molecular docking analysis was employed to elucidate the potential mechanism of VD supplementation on the progression of IgAN.

**Results:**

During a median follow-up of 4.3 years (interquartile range (IQR): 3.3–5.9 years), a total of 92 (10.6%) patients experienced MAKE. Cumulative renal outcomes were significantly higher in patients with lower baseline and TWA 25(OH)D levels. The multivariate Cox regression analyses indicated that TWA 25(OH)D level was an independent determinant for MAKE in IgAN after adjusting for important confounders. Moreover, it showed reliable predictive performance in risk stratification of MAKE, with the optimal predictive cut-off value of 44.8 nmol/L. Accordingly, a significant linear association was observed between TWA 25(OH)D and the risk of MAKE. Reclassification further confirmed the consistency of the overall findings. Furthermore, in addition to routinely used clinical parameters, the TWA 25(OH)D-based model demonstrated strong risk-prediction power, verified internally, and showed satisfactory efficacy and significant net advantages. Moreover, VD treatment may improve prognosis by regulating the processes of cell chemotaxis, inflammatory response, and defense response through targeting the expressions OF NFKB1 and NR4A1 in proximal tubule cells in IgAN.

**Conclusion:**

Our findings provide a more comprehensive insight into VD in IgAN and strengthen the efficacy of VD supplementation in IgAN. The long-term maintenance of optimal VD levels from early in life might be associated with reduced future risk of kidney progression in IgAN.

## 1 Introduction

The IgA nephropathy (IgAN) has transcended its status as a relatively common manifestation of glomerulonephritis to emerge as a causative agent of kidney failure in a disproportionately large proportion of individuals under the age of 40. Its clinical presentation is inherently varied and multifaceted, yet it has become increasingly recognized as a leading contributor to progressive renal pathology, with incidence rates exceeding 20–50% among affected patients (1, 2). The clinical manifestations of IgAN are diverse and have increasingly become a significant factor in the development of progressive renal disease. This condition accounts for approximately 20–50% of cases, affecting approximately one-third of patients over an extended period of 10–20 years (3). Consequently, the Kidney Disease Improving Global Outcomes (KDIGO) clinical practice guidelines recommend stratified risk assessment for each individual, thereby facilitating personalized and optimized therapeutic interventions (4, 5). The advent of a novel predictive model has revolutionized our understanding of IgAN, marking a significant paradigm shift in the traditional approach to risk assessment. By integrating an array of critical patient characteristics, including demographic factors such as ethnicity, sex, systolic blood pressure (SBP), proteinuria level, estimated glomerular filtration rate (eGFR), and histopathological features, into the International IgAN Network risk prediction score, clinicians now have a more comprehensive framework for estimating the likelihood of disease progression (4, 6). Despite significant advancements in our understanding of IgAN, uncertainty persists regarding its progression. The International IgAN Network risk prediction score is a valuable tool in stratifying patients at high and low risk, but controversy persists regarding optimal therapeutic strategies for individuals with low-risk profiles (7). In contrast to initial expectations about the chronic nature of IgAN, patients can exhibit considerable variability in their disease progression. This disparity arises from fluctuations in course, with some individuals responding well to short-term interventions but ultimately succumbing to relapse after prolonged periods (2, 7). Given this context, it is increasingly recognized as valuable to explore novel markers for assessing IgAN disease severity and contributing to risk stratification dynamically during follow-up.

Vitamin D (VD), a steroid hormone with diverse physiological activities, is known to play a critical role in maintaining normal mineral homeostasis and bone health (8). Concurrently, growing evidence from animal studies and clinical observations has revealed a connection between VD and kidney diseases, underscoring its potential impact on renal function (9). The synthesis of 25-hydroxyvitamin D [25(OH)D] by the enzyme 25-hydroxylase catalysis is a crucial metabolic pathway that enables the assessment of VD status in clinical settings and epidemiological studies (10). VD deficiency is a common condition affecting a substantial proportion of patients with chronic kidney disease (CKD), exceeding the prevalence of approximately 80% among non-dialysis CKD patients (11). Furthermore, it has been posited that lower serum concentrations of 25(OH)D are correlated with a worsening of renal function and an increased susceptibility to mortality in patients with CKD, even among those who do not require dialysis (9).

Studies on Chinese patients with IgAN have shown a correlation between VD levels and the manifestations of IgAN. VD deficiency is associated with a more pronounced disease severity. Additionally, supplementation with VD combined with a renin–angiotensin system (RAAS) inhibitor has been proven to reduce proteinuria in patients with IgAN (12–16). A most intriguing finding has emerged from a recent investigation into the relationship between VD levels and the clinical manifestations of IgAN in Indian patients. Contrary to expectations, data from this study revealed no significant correlation between VD deficiency and disease profile or renal outcomes (17). A finding that revealed a significant gap in our understanding has prompted a high demand for further elucidation about the relationship between VD levels and IgAN pathology. Furthermore, whether VD supplementation is an effective treatment strategy for IgAN requires further investigation to shed light on key decisions regarding patient care and resource allocation.

This investigation aimed to elucidate the significance of baseline and dynamic changes in serum 25(OH)D levels in relation to clinical features and outcomes of major adverse kidney events (MAKE) in patients with IgAN. By leveraging a large sample size and long-term follow-up data, this study sought to provide insight into the potential role of VD supplementation in mitigating kidney damage. Further investigation was conducted using integrated network pharmacology analysis, molecular docking, and single-cell RNA sequencing (scRNA-seq) to explore the mechanisms by which VD treatment may slow down kidney disease progression in IgAN pathology.

## 2 Materials and methods

### 2.1 Subjects

The retrospective cohort study examined patients with primary IgAN who had undergone renal biopsy within the Department of Nephrology at the First Affiliated Hospital of Nanjing Medical University between 1 January 2014 and 31 December 2019. The definition of IgAN was according to the Kidney Disease: Improving Global Outcomes (KDIGO) 2021 Clinical Practice Guideline for the Management of Glomerular Diseases (5). The exclusion criteria included: (1) patients with estimated glomerular filtration rate (eGFR) < 15 mL/min/1.73 m^2^ or with renal replacement treatment; (2) patients with other systemic diseases involving the kidney, such as allergic purpura, vasculitis, or systemic lupus erythematosus; (3) patients with incomplete baseline data, such as 25(OH)D; (4) lost to follow-up. Ultimately, 866 patients with IgAN were included in the study to form the baseline cohort for 25(OH)D. Among them, 430 patients with complete data on time-weighted average (TWA) serum 25(OH)D levels were selected to form the follow-up TWA cohort. This study was approved by the Ethics Committee with The First Affiliated Hospital of Nanjing Medical University, which granted a waiver of informed consent, and was conducted in accordance with the 1964 Helsinki Declaration and its subsequent amendments or comparable ethical standards.

### 2.2 Clinical and laboratory parameters

Baseline demographics, complications, comorbidities, treatment, laboratory parameters, pathological data at biopsy, and outcomes were collected in detail from the electronic medical records of enrolled patients. The eGFR for all patients was calculated using the Chronic Kidney Disease Epidemiology Collaboration (CKD-EPIcr) formula (18). The TWA value was derived as an aggregate area under the curve divided by the cumulative time exposure for each patient. The area under the curve was measured as an integrated expression over time using a positive incremental method, without imputation for missing time points. The calculated formula is as follows: TWA serum 25(OH)D = {[(X1 + X2) (T2 − T1) + (X2 + X3) (T3 − T2) + … + (Xn − 1 + Xn) (Tn − Tn − 1)]/[2 × (Tn − T1)]}, where Tn is nth time point and Xn is the serum 25(OH)D level at Tn (19). Routine examination of every renal biopsy specimen was performed using light microscopy, electron microscopy, and immunofluorescence and evaluated by two nephropathologists. All kidney biopsy slides were scored according to Lee’s pathological grade, Haas classification grade, and the updated Oxford Classification (MEST-C) (20–22). Any scoring differences between the two pathologists were repeatedly reviewed until a consensus was obtained.

### 2.3 Study outcomes

The primary endpoint was the major adverse kidney events (MAKE), a composite definition encompassing death from any cause, or dependence on renal replacement therapy up to October 2023 (cut-off date), or the doubling of serum creatinine (D-Scr) from baseline. For patients without outcomes, the end date was defined as the time of the most recent hospitalization or clinic visit. The follow-up period for each patient was calculated as the number of days between the patient’s discharge date following a renal biopsy and the end date itself.

### 2.4 Collection of IgAN-targeted genes

The IgAN-targeted genes were identified by integrating databases through keyword searches for “IgA Nephropathy” and then taking the intersection. Four databases include the DisGeNET database,^1^ the GeneCards database (LifeMap Sciences),^2^ the DrugBank database,^3^ and the OMIM database.^4^

### 2.5 Potential targets of 25(OH)D treatment in IgAN

To evaluate the biological information of 25(OH)D, the two-dimensional (2D) and 3D molecular structures of 25(OH)D were obtained from the PubChem database.^5^ Next, the potential targets of 25(OH)D were predicted using the SwissTargetPrediction database,^6^ the SuperPred database,^7^ and the DrugBank database. After that, all targets were converted into gene symbols standardized through the Uniprot database.^8^

### 2.6 Transcriptomics data acquisition and processing

RNA-seq profiling and scRNA-seq data were downloaded from the Gene Expression Omnibus (GEO) database.^9^ The GSE175759 dataset included data on kidney samples from patients with 46 IgAN and 22 healthy controls on the GPL16791 platform [Illumina HiSeq 2500 (*Homo sapiens*)]. The scRNA-seq data from four patients with IgAN and one healthy control sample were obtained from the GSE171314 dataset on GPL20795 [HiSeq X Ten (*Homo sapiens*)]. For scRNA-seq data, “DoubletFinder” was used to remove the potential doublets. “Seurat” was used to standardize the expression of filtered samples. Cells were filtered out with a threshold of the ratio of mitochondrial genes ≤50% and ribosomal genes <10%. Genes expressed in >5 cells and cells with at least 500 genes were retained. An integrated dataset was created by using the “Harmony” data integration method.

### 2.7 Dimensionality reduction and cell annotation

The integrated data were then subjected to principal component analysis (PCA), and the dimensions of the top 50 PCs were reduced using the Uniform Manifold Approximation and Projection for Dimension Reduction (UMAP) algorithm to obtain principal clusters. Finally, the cells were labeled and clustered according to their expression of the marker gene.

### 2.8 Differentially expressed genes identification

For the GSE175759 dataset, we modeled a negative binomial distribution, and differentially expressed genes (DEGs) were identified as those with a Benjamini–Hochberg-adjusted false discovery rate <0.05 and |fold change| > 1 using the “edgeR” v4 package in R.^10^ For the GSE171314 dataset, the “FindMarkers” function in Seurat was used to compare the gene expression between IgAN and healthy control groups, with a significant FDR cut-off of 0.05 and log(fold change) of 0.5.

### 2.9 Identification of gene signatures in 25(OH)D and IgAN

The potential targets of 25(OH)D were matched with IgAN-related genes to obtain the core targets. The Venn diagram was drawn using the “VennDiagram” R package.^11^ Then, we selected the core targets that were significantly differentially expressed in IgAN samples. To explore the potential roles of these target genes, a biological analysis of these shared genes was performed by “clusterProfiler” and “org.Hs.eg.db” packages in R software. The biological process of GO analysis was focused. The adjusted *p*-value <0.05 was considered significant.

### 2.10 Molecular docking

Proteins corresponding to the core target genes were selected to dock with the 25(OH)D and 1, 25(OH)D molecules to verify their affinity. Crystal structures of proteins were downloaded from the PDB database.^12^ The protein structures were imported into PyMOL 2.2.0 software (The PyMOL Molecular Graphics System, Schrödinger, LLC).^13^ for modification, including the removal of water molecules, separation of ligands, and addition of hydrogen atoms. AutoDockTools 1.5.6 software [The Scripps Research Institute, (TSRI)]^14^ was used to add a charge to the protein molecule and set up a docking grid box centered on the molecule. Molecular docking was performed using AutoDock 4.27 software [The Scripps Research Institute, (TSRI)] with a genetic algorithm. Finally, after analyzing the binding energy of the molecule, selecting the conformation with the lowest binding energy, and observing the formation of hydrogen bonds, we created a binding diagram using PyMOL 2.2.0.

### 2.11 Statistical analysis

All statistical analyses were conducted using R statistical software [R Core Team (2024)].^15^ Data were presented as mean ± standard deviation (SD), median, and interquartile range or percentage. Comparisons between groups were made using one-way analysis of variance (ANOVA), the Kruskal–Wallis test, or the *χ*^2^ test as appropriate. Kaplan–Meier analysis and the log–rank test were used to assess MAKE differences, and the surv_cutpoint function was used to determine the optimal cut-off value for the 25(OH)D level. Receiver operating characteristic (ROC) curve analyses were performed to characterize the predictive accuracy of 25(OH)D level, TWA 25(OH)D level, eGFR, and proteinuria. The DeLong test was then used to compare the ROC curves of different parameters. Spearman correlations were calculated to characterize the associations between baseline characteristics and serum 25(OH)D level or TWA 25(OH)D level. Hazard ratios for 25(OH)D and TWA 25(OH)D levels with MAKE were estimated using Cox proportional hazards regression models with follow-up time. Subgroup analyses were performed for age, sex, serum albumin, SBP, body mass index (BMI), use of RAAS inhibitors, steroids, and/or immunosuppressants, and VD supplementation in the association between TWA 25(OH)D level and MAKE. Cox proportional hazards regression was used to establish the prediction model. The backward step method was used to select variables for the final model. The performance of the prediction model was assessed by discrimination, calibration, and decision curve analysis (DCA). Discrimination was addressed using the concordance index (C-index) and area under the receiver operating characteristic curve (AUC). Calibration was determined by a visual calibration plot comparing the predicted and actual probability of MAKE in IgAN. Then, DCA was used to determine the clinical benefit of the models proposed by the “rmda” package;^16^ *p*-value <0.05 was considered statistically significant.

## 3 Results

### 3.1 Basic characteristics

**Figure 1:**
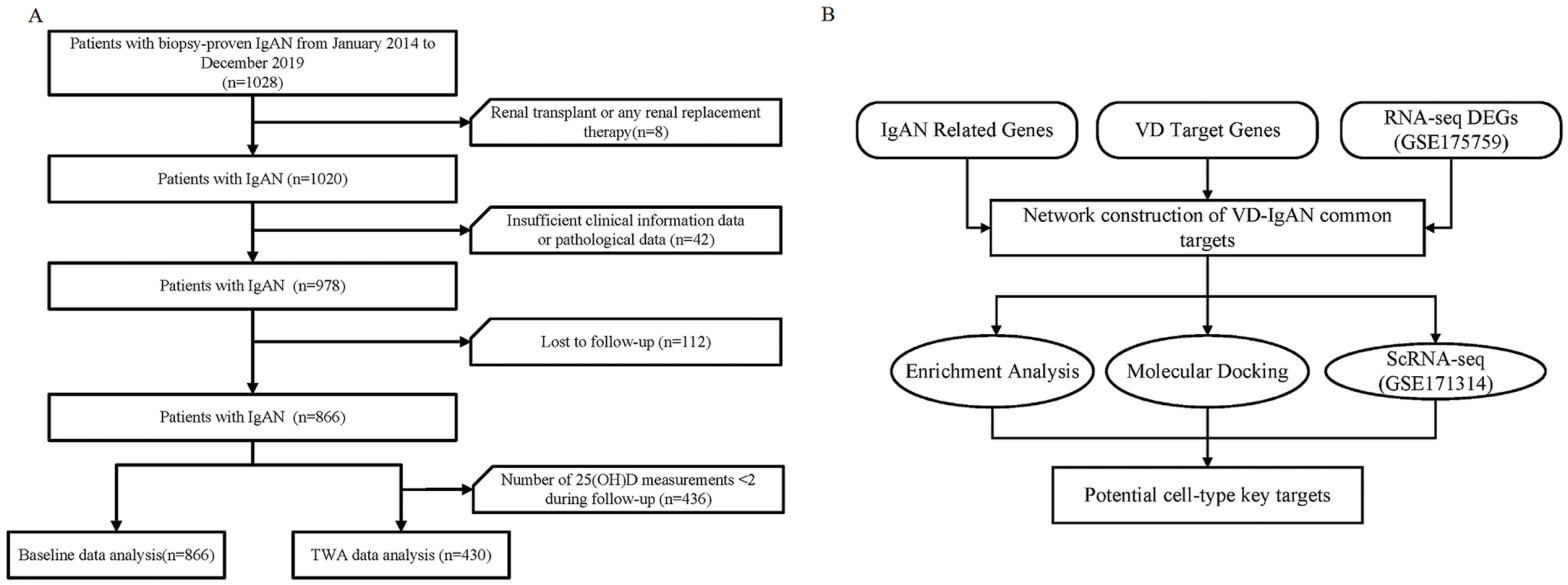
Flowchart of the study. **(A)** Flowchart of study participants. **(B)** Flowchart of the integrative analysis of potential VD targets in IgAN. IgAN, IgA nephropathy; TWA, time-weighted average; VD, vitamin D; 25(OH)D, 25-hydroxy vitamin D; DEGs, differentially expressed genes; ScRNA-seq, single cell RNA sequencing.

Overall, 866 biopsy-proven IgAN patients [39 (IQR29, 49) years, 47% male] who met the enrollment criteria were ultimately included in the present study (Figures 1A,B). At baseline, the median urinary protein was 793.19 (320.21, 1,806.93) mg/d and eGFR was 97 mL/min/1.73 m^2^ (IQR: 68, 113). During a median follow-up of 4.3 years (IQR: 3.3–5.9 years), a total of 92 (10.6%) patients experienced MAKE, including 3 (0.3%) deaths, 58 (6.7%) who received kidney replacement therapy, and 31 (3.6%) D-Scr. According to the incidence of MAKE, patients were divided into two groups as summarized in Table 1. Patients with a composite of the MAKE tended to have a larger burden of concomitant diseases, including diabetes mellitus, hypertension, cardiovascular diseases, and more severe CKD stages compared with those in the non-MAKE group (*p* < 0.05). Moreover, higher levels of BMI, SBP, diastolic blood pressure (DBP), triglyceride (TG), low-density lipoprotein cholesterol (LDL-C), D-dimer, fibrinogen, and C4, while lower levels of high-density lipoprotein cholesterol (HDL-C), hemoglobin, activated partial thromboplastin time (APTT), and serum IgG were observed in patients with poor prognosis (*p* < 0.05, vs. non-MAKE). Regarding kidney function, patients with adverse outcomes had higher levels of serum creatinine (Scr), blood urea nitrogen (BUN), uric acid, and proteinuria, as well as lower levels of retinol binding protein (RBP), but similar levels of eGFR and serum albumin compared to those with non-MAKE (*p* < 0.05). Regarding the pathological characteristics, patients in the MAKE group were more likely to have more advanced Lee’s grade, Hass’s grade, and more severe S, T, and C scores (*p* < 0.05 vs. non-MAKE). Furthermore, VD supplementation was significantly lower in patients with MAKE compared to those in the non-MAKE group (*p* < 0.05).

**Table 1:** Baseline clinicopathological characteristics of the entire population stratified by the primary endpoint.

Parameter	Total (*n* = 866)	MAKE (*n* = 92)	Non-MAKE (*n* = 774)	*p*-value
Age (years)	39 (29, 49)	41 (32, 51)	38 (29, 49)	0.039
	40 ± 13	43 ± 14	39 ± 12	
Sex (male/female)	407/459	49/43	358/416	0.245
MAKE, No. (%)	92 (10.6)	92 (100)	0 (0)	<0.001
Death, No. (%)	3 (0.3)	3 (3.3)	0 (0)	<0.001
New kidney replacement therapy, No. (%)	58 (6.7)	58 (63)	0 (0)	<0.001
D-Scr, No. (%)	31 (3.6)	31 (34)	0 (0)	<0.001
CKD stage (1/2/3a/3b/4)	507/188/75/60/36	16/20/12/19/25	491/168/63/41/11	<0.001
Comorbid disease				
Hypertension (%)	283 (33)	44 (48)	239 (31)	0.002
Cardiovascular diseases (%)	11 (1.3)	5 (5.4)	6 (0.008)	0.003
Diabetes mellitus (%)	42 (4.8)	7 (7.6)	35 (4.5)	0.033
Clinical parameter				
BMI (kg/m^2^)	23.7 (21.3, 26.5)	24.8 (22.0, 27.3)	23.6 (21.1, 26.3)	0.007
	23.5 ± 5.6	25.1 ± 3.8	23.3 ± 5.8	
SBP (mm Hg)	128 (118, 140)	134 (123, 151)	128 (118, 139)	<0.001
	132 ± 54	154 ± 15	129 ± 17	
DBP (mm Hg)	82 (74, 90)	86 (78, 96)	82 (74, 90)	0.004
	83 ± 13	87 ± 16	82 ± 12	
Laboratory parameter				
Urinary protein (mg/day)	793 (320, 1,807)	2,210 (1,043, 4,112)	700 (280, 1,578)	<0.001
	1,581 ± 2,225	2,695 ± 2,232	1,442 ± 2,186	
eGFR (mL/min/1.73 m^2^)	97 (68, 113)	47 (28, 74)	100 (76, 115)	<0.001
	89 ± 30	56 ± 32	93 ± 27	
BUN (mmol/L)	5.3 (4.4, 6.7)	7.7 (6.2, 10.8)	5.2 (4.2, 6.5)	<0.001
	5.9 ± 2.7	9.0 ± 4.9	5.6 ± 2.0	
Scr (μmol/L)	76.3 (61.1, 104.3)	133.7(92.2, 225.1)	73.6 (60.0, 98.0)	<0.001
	92.6 ± 50.8	155.9 ± 84.1	84.9 ± 38.7	
Uric acid (μmol/L)	356 (289, 438)	432 (347, 495)	349 (283, 431)	<0.001
	368 ± 111	433 ± 132	360 ± 105	
Serum albumin (g/L)	38.9 (35.4, 42.3)	35.3 (29.2, 39.1)	39.1 (35.8, 42.6)	<0.001
	38.3 ± 7.3	35.9 ± 8.9	38.6 ± 7.0	
Serum 25(OH)D (nmol/L)	37.4 (25.8, 51.0)	30.2 (19.6, 42.6)	37.9 (26.3, 51.6)	<0.001
	39.3 ± 18.8	32.9 ± 16.2	40.1 ± 19.0	
FBG (mmol/L)	4.7 (4.4, 5.2)	4.7 (4.3, 5.1)	4.7 (4.4, 5.2)	0.629
	4.9 ± 1.0	4.8 ± 1.0	4.9 ± 1.0	
TG (mmol/L)	1.27 (0.91, 1.87)	1.65 (1.15, 2.42)	1.23 (0.88, 1.80)	<0.001
	1.65 ± 1.36	2.24 ± 2.05	1.57 ± 1.23	
TC (mmol/L)	4.58 (3.95, 5.44)	4.78 (3.99, 6.02)	4.57 (3.94, 5.41)	0.122
	4.93 ± 1.68	5.07 ± 1.49	4.91 ± 1.70	
LDL-C (mmol/L)	3.00 (2.52, 3.71)	3.28 (2.61, 4.07)	2.99 (2.51, 3.66)	0.047
	3.25 ± 1.22	3.41 ± 1.08	3.23 ± 1.23	
HDL-C (mmol/L)	1.11 (0.93, 1.33)	1.05 (0.87, 1.23)	1.12 (0.94, 1.33)	0.019
	1.19 ± 0.78	1.10 ± 0.31	1.21 ± 0.82	
Hemoglobin (g/L)	132 (119, 145)	119 (105, 135)	134 (122, 146)	<0.001
	132 ± 20	119 ± 20	133 ± 19	
WBC (×10^9^/L)	6.6 (5.5, 7.8)	6.9 (5.6, 8.1)	6.6 (5.5, 7.8)	0.322
	6.9 ± 2.3	6.9 ± 1.8	6.9 ± 2.4	
Neutrophil (×10^9^/L)	4.1 (3.3, 5.1)	4.5 (3.4, 5.5)	4.1 (3.2, 5.0)	0.091
	4.5 ± 2.9	4.58 ± 1.7	4.49 ± 3.0	
Neutrophil (%)	62 (56, 68)	64 (58, 71)	62 (56, 68)	0.016
	62 ± 10	65 ± 10	62 ± 10	
Lymphocyte (×10^9^/L)	1.9 (1.5, 2.3)	1.7 (1.4, 2.1)	1.9 (1.5, 2.3)	0.056
	1.9 ± 0.7	1.8 ± 0.6	1.9 ± 0.7	
Lymphocyte (%)	29 (23, 34)	27 (21, 31)	29 (24, 34)	0.005
	29 ± 8	27 ± 9	29 ± 8	
Monocyte (×10^9^/L)	0.39 (0.30, 0.50)	0.39 (0.29, 0.48)	0.39 (0.30, 0.50)	0.452
	0.42 ± 0.17	0.39 ± 0.13	0.42 ± 0.17	
Monocyte (%)	5.9 (4.8, 7.1)	5.6 (4.8, 6.5)	5.9 (4.8, 7.2)	0.053
	6.2 ± 2.6	5.8 ± 1.8	6.3 ± 2.7	
Platelet (×10^9^/L)	216 (177, 258)	211 (165, 258)	218 (179, 258)	0.433
	221 ± 67	219 ± 77	222 ± 66	
PT (s)	11.8 (11.4, 12.3)	11.8 (11.4, 12.2)	11.8 (11.4, 12.3)	0.67
	11.9 ± 0.8	11.8 ± 0.6	11.9 ± 0.8	
INR	1.03 (0.98, 1.07)	1.03 (0.98, 1.06)	1.03 (0.99, 1.07)	0.439
	1.05 ± 0.60	1.02 ± 0.05	1.05 ± 0.64	
APTT (s)	27.8 (25.9, 29.7)	26.8 (25.0, 28.9)	27.9 (26, 29.8)	0.006
	27.9 ± 3.4	27.2 ± 3.8	28.0 ± 3.3	
TT (s)	18.2 (17.5, 18.8)	18.1 (17.5, 18.7)	18.2 (17.5, 18.9)	0.674
	18.2 ± 1.4	18.2 ± 1.2	18.2 ± 1.4	
D-dimer (mg/L)	0.25 (0.16, 0.43)	0.36 (0.21, 0.56)	0.24 (0.15, 0.41)	<0.001
	0.81 ± 5.94	0.73 ± 1.65	0.82 ± 6.26	
Fibrinogen (g/L)	2.80 (2.34, 3.44)	3.30 (2.97, 3.75)	2.72 (2.3, 3.33)	<0.001
	3.00 ± 1.06	3.44 ± 0.93	2.94 ± 1.06	
IgA (g/L)	3.1 (2.4, 4.1)	3.0 (2.4, 3.8)	3.1 (2.4, 4.2)	0.333
	3.1 ± 1.1	2.9 ± 0.9	3.1 ± 1.1	
IgG (g/L)	11.6 (9.6, 14.5)	10.8 (9.1, 13.4)	11.7 (9.7, 14.8)	0.022
	11.3 ± 3.1	10.8 ± 3.1	11.3 ± 3.1	
IgM (g/L)	1.01 (0.72, 1.43)	0.96 (0.57, 1.48)	1.02 (0.73, 1.42)	0.125
	1.16 ± 0.65	1.11 ± 0.67	1.16 ± 0.64	
C3 (g/L)	1.08 (0.94, 1.29)	(0.95, 1.32)	1.08 (0.94, 1.26)	0.56
	14.03 ± 34.44	12.37 ± 32.26	14.24 ± 34.72	
C4 (g/L)	0.26 (0.20, 0.32)	0.28 (0.24, 0.35)	0.25 (0.20, 0.32)	0.001
	3.47 ± 8.94	3.44 ± 9.30	3.48 ± 8.90	
RBP (mg/L)	50.3 (38.7, 64.0)	65.9 (51.4, 81.0)	49.4 (38.0, 62.3)	<0.001
	54.7 ± 23.2	66.6 ± 24.7	53.2 ± 22.6	
Pathological parameter				
Lee (1/2/3/4/5)	32/265/443/100/26	3/15/38/27/9	29/250/405/73/17	<0.001
Haas (1/2/3/4/5)	97/401/244/92/32	8/34/17/20/13	89/367/227/72/19	<0.001
M (0/1)	649/217	76/16	573/201	0.095
E (0/1)	848/18	89/3	759/15	0.427
S (0/1)	170/696	8/84	162/612	0.008
T (0/1/2)	746/96/24	71/17/4	675/79/20	0.026
C (0/1/2)	479/272/115	51/22/19	428/250/96	0.048
Medications				
Vitamin D supplementation (%)	589 (68)	49 (52)	540 (70)	<0.001
Dosage of vitamin D supplementation (%)				<0.001
0–400 IU/day	369 (43)	56 (60)	313 (40)	
400–1,000 IU/day	313 (36)	28 (30)	285 (37)	
>1,000 IU/day	186 (21)	10 (11)	176 (23)	
RAAS inhibitor (%)	220 (25)	19 (21)	201 (26)	0.373
Steroids and/or immunosuppressants (%)	422 (49)	38 (41)	384 (50)	0.162

BMI, Body mass index; SBP, systolic blood pressure; DBP, diastolic blood pressure; eGFR, estimated glomerular filtration rate; BUN, blood urea nitrogen; Scr, serum creatine; 25(OH)D, 25-hydroxy vitamin D; FBG, fasting blood glucose; HbAc1, glycosylated hemoglobin; TG, triglyceride; TC, total cholesterol; LDL-C, low-density lipoprotein cholesterol; HDL-C, high-density lipoprotein cholesterol; WBC, white blood cell; PT, prothrombin time; INR, international normalized ratio; APTT, activated partial thromboplastin time; TT, thrombin time; URBC, urinary red blood cell; IgA, Immunoglobulin A; IgG, Immunoglobulin G; C3, complement 3; C4, complement 4; RBP, retinol binding protein; RAAS, renin–angiotensin–aldosterone system; MAKE, major adverse kidney events; D-Scr, doubling of serum creatinine level.

Data were presented as the mean ± standard deviation (SD), the median with interquartile range, or counts and percentages. A two-tailed *p* < 0.05 was considered statistically significant.

### 3.2 Association of baseline 25(OH)D with clinicopathological parameters and MAKE

**Figure 2:**
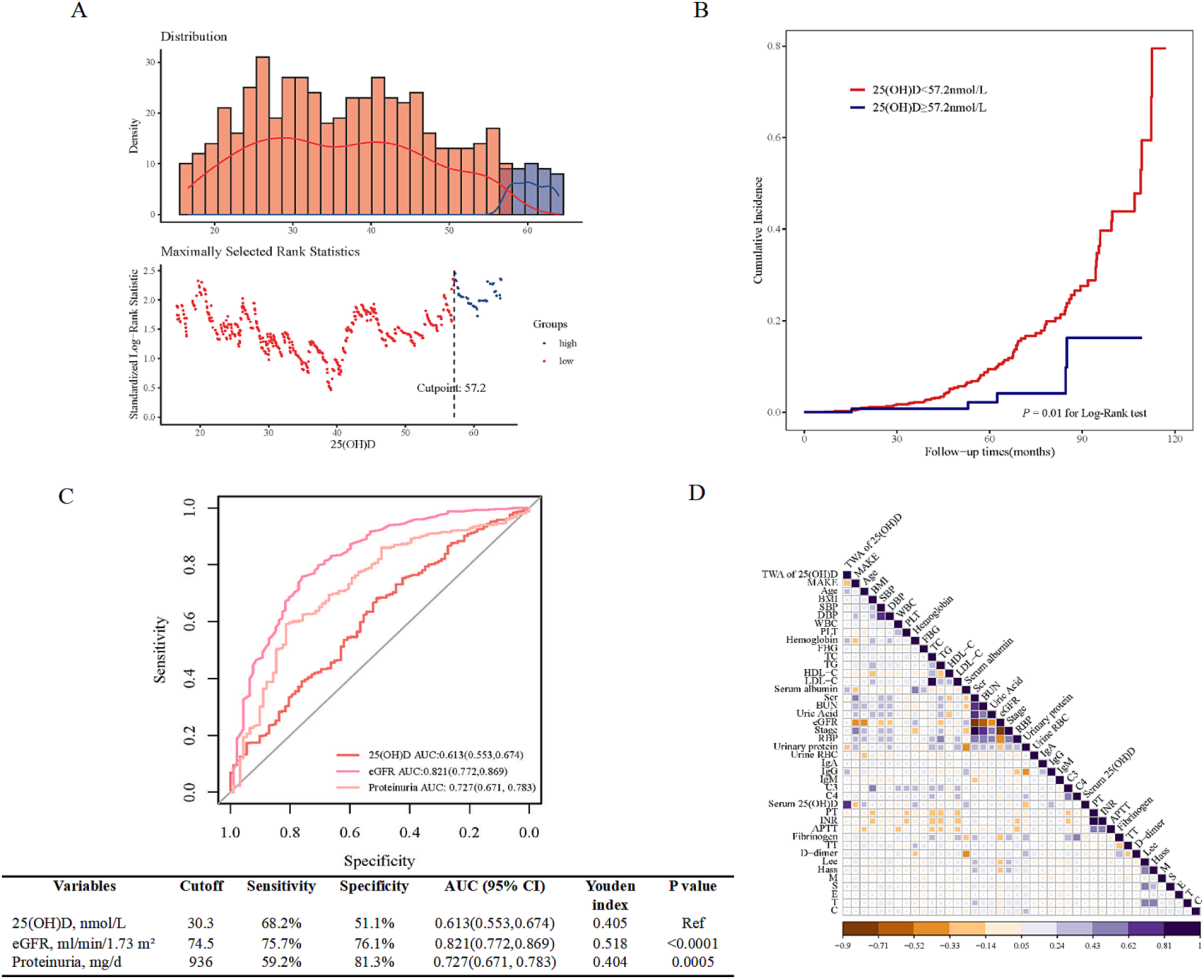
The prognostic ability of baseline 25(OH)D for MAKE. **(A)** The optimal cut-off point for the baseline 25(OH)D is 57.2 nmol/L, as determined using the surv_cutpoint function of the R package survminer. **(B)** The comparison of the cumulative incidence of MAKE by Kaplan–Meier analysis between the high-25(OH)D D group (>the cut-off value) and the low-25(OH)D group (≤the cut-off value). **(C)** ROC curves for the diagnostic performance of baseline 25(OH)D, eGFR, and 24 h proteinuria for discriminating different MAKE risk categories. **(D)** The correlation between 25(OH)D and clinical parameters. MAKE, major adverse kidney events; 25(OH)D, 25-hydroxy vitamin D; eGFR, estimated glomerular filtration rate; TWA, time-weighted average; BMI, body mass index; SBP, systolic blood pressure; DBP, diastolic blood pressure; WBC, white blood cell; PLT, platelet; FBG, fasting blood glucose; TC, total cholesterol; TG, triglyceride; HDL-C, high-density lipoprotein cholesterol; LDL-C, low-density lipoprotein cholesterol; Scr, serum creatine; BUN, blood urea nitrogen; RBP, retinol binding protein, urine RBC, urinary red blood cell; IgA, immunoglobulin A; IgG, immunoglobulin G; C3, complement 3; C4, complement 4; PT, prothrombin time; INR, international normalized ratio; APTT, activated partial thromboplastin time; TT, thrombin time.

**Figure 3:**
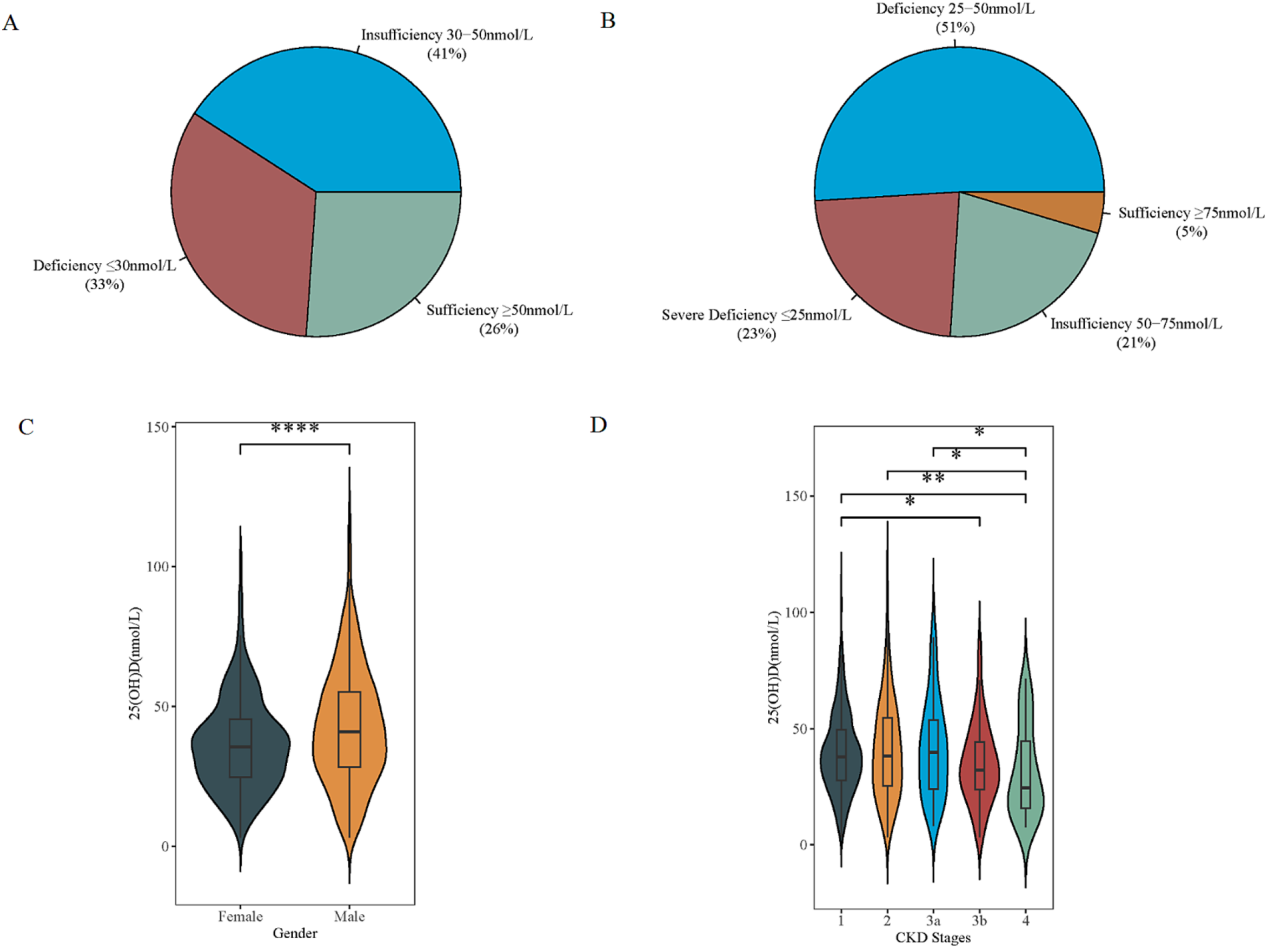
Distribution of baseline VD status in IgAN. **(A)** Four categories of baseline VD status according to the Endocrine Society clinical practice (ESC) guidelines and the UK-based Scientific Advisory Committee on Nutrition (SACN). **(B)** Three categories according to the Institute of Medicine (IOM). **(C,D)** Significant difference was obtained in VD levels between males and females, and at different stages of CKD, respectively. *p < 0.05 **p < 0.01, ***p < 0.001, and ****p < 0.0001. VD, vitamin D; 25(OH)D, 25-hydroxyvitamin D; CKD, chronic kidney disease.

Based on the results that serum 25(OH)D level significantly discriminated between patients with and without the incidence of MAKE, the optimal cut-off value of 25(OH)D for the risk of MAKE was further analyzed by the surv_cutpoint function of the R package survminer (Figure 2A).^17^ Accordingly, stratified by the cut-off value, the Kaplan–Meier analysis curves revealed that the incidence of MAKE was significantly higher in the high-25(OH)D group (≥57.2 nmol/L) compared to the low-25(OH)D group (<57.2 nmol/L) (log-rank test *p* = 0.01, Figure 2B). Additionally, ROC curve analysis revealed that the AUC of 25(OH)D for differentiating the risks of MAKE was 0.613 (sensitivity = 68.2%; specificity = 51.1%; Figure 2C). eGFR showed the largest AUC of 0.821 for differentiating MAKE from non-MAKE [sensitivity = 75.7%, specificity = 76.1%, *p* < 0.0001 vs. 25(OH)D], which was followed by 24 h proteinuria [AUC = 0.727, sensitivity = 59.2%, specificity = 81.3%, *p* = 0.0005 vs. 25(OH)D]. The basic clinicopathological characteristics between the two groups were compared (Supplementary Table S1). The correlation between serum 25(OH)D levels and clinicopathological characteristics, as determined by further Spearman correlation analyses, is presented in Figure 2D. There was a positive relationship between baseline 25(OH)D and hemoglobin (*r* = 0.25, *p* < 0.001), serum albumin (*r* = 0.35, *p* < 0.001), serum IgG (*r* = 0.31, *p* < 0.001), and a negative relationship between 25(OH)D and white blood cell (*r* = −0.12, *p* = 0.007), platelet (*r* = −0.16, *p* < 0.001), serum total cholesterol (TC) (*r* = −0.15, *p* < 0.001), 24 h urinary protein (*r* = −0.35, *p* < 0.001), fibrinogen (*r* = −0.20, *p* < 0.001), and D-dimer (*r* = −0.13, *p* < 0.001). Furthermore, in accordance with the Endocrine Society clinical practice (ESC) and the UK-based Scientific Advisory Committee on Nutrition (SACN) guidelines, only 5 and 21% IgAN patients showed sufficient (≥75 nmol/L) and insufficient baseline 25(OH)D levels (50–75 nmol/L), with 23% exhibiting 25(OH)D severe deficiency (<25 nmol/L) (Figure 3A). Meanwhile, according to the Institute of Medicine (IOM) guideline, the proportions of patients with sufficient (≥50 nmol/L), insufficient (30–50 nmol/L), and deficiency (<30 nmol/L) 25(OH)D levels were 26%, 41%, and 33%, respectively (Figure 3B). Moreover, male patients had significantly higher 25(OH)D levels compared to females (Figure 3C). And 25(OH)D level obviously decreased across increasing CKD stages (G1 vs. G3b: *p* = 0.035; G1 vs. G4: *p* < 0.01; G2 vs. G4: *p* = 0.014; G3a vs. G4: *p* = 0.017, Figure 3D).

### 3.3 Risk assessment of TWA 25(OH)D for the incidence of MAKE

**Figure 4:**
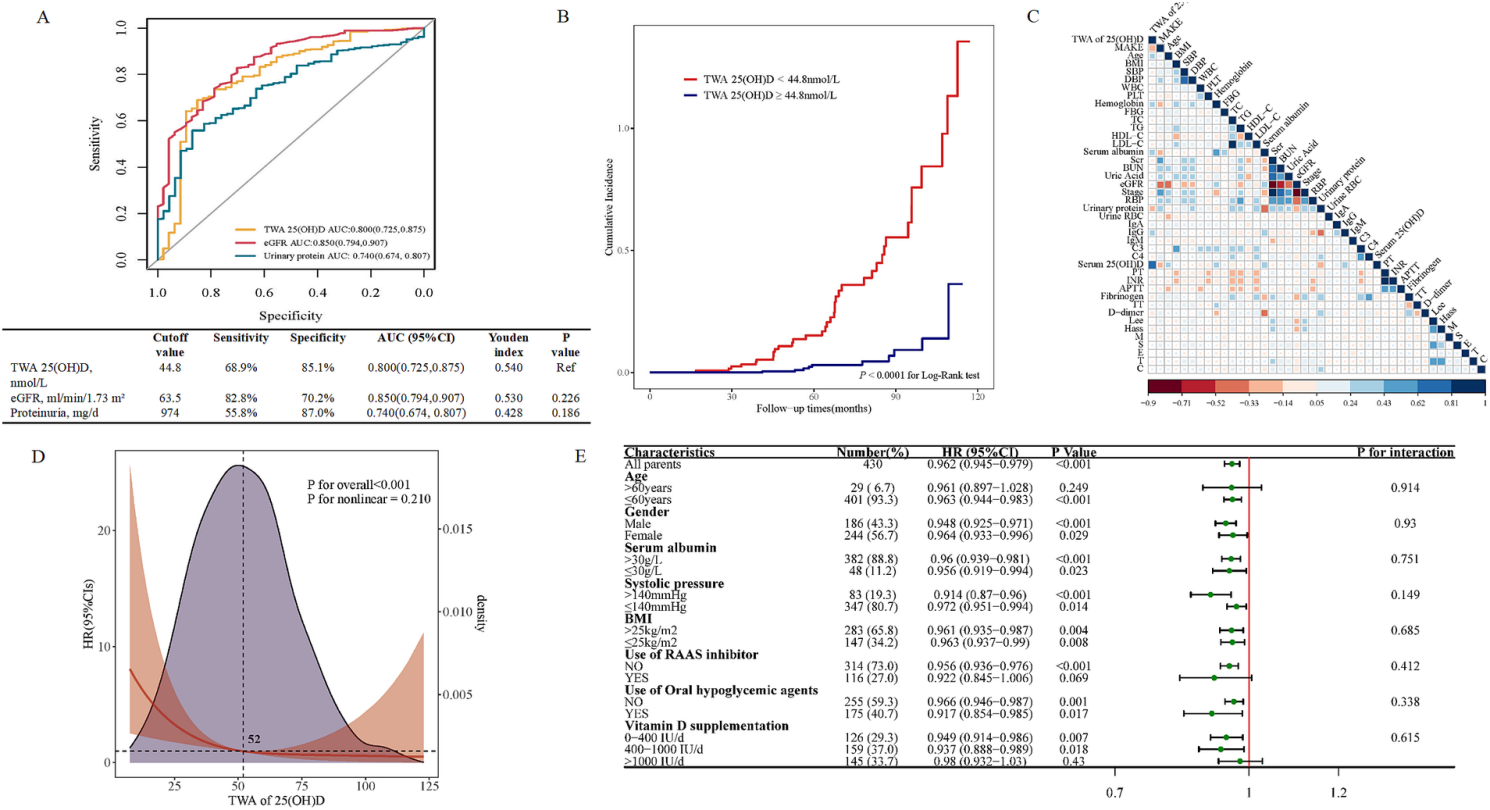
The prognostic ability of TWA 25(OH)D for MAKE. **(A)** ROC curves for the diagnostic performance of TWA 25(OH)D, eGFR, and proteinuria for discriminating different MAKE risk categories. **(B)** The comparison of the cumulative incidence of MAKE by Kaplan–Meier analysis between the high-TWA 25(OH)D group (>the cut-off value) and the low-TWA 25(OH)D group (≤the cut-off value). **(C)** The correlation between TWA 25(OH)D and clinical parameters. **(D)** Association of TWA 25(OH)D with HR of MAKE by a restricted cubic spline curve. The restricted cubic spline curve was plotted using three default knots. The hazard ratio was adjusted for baseline eGFR, 24 h urinary protein, TG, C4, and lymphocyte count. The *p-*value for the overall association was <0.001. **(E)** Forest plot of TWA 25(OH)D and MAKE in the subgroups. HRs were adjusted for baseline eGFR, 24 h urinary protein, TG, C4, and lymphocyte count. MAKE, major adverse kidney events; 25(OH)D, 25-hydroxy vitamin D; eGFR, estimated glomerular filtration rate; TWA, time-weighted average; BMI, body mass index; SBP, systolic blood pressure; DBP, diastolic blood pressure; WBC, white blood cell; PLT, platelet; FBG, fasting blood glucose; TC, total cholesterol; TG, triglyceride; HDL-C, high-density lipoprotein cholesterol; LDL-C, low-density lipoprotein cholesterol; Scr, serum creatine; BUN, blood urea nitrogen; RBP, retinol binding protein, urine RBC, urinary red blood cell; IgA, immunoglobulin A; IgG immunoglobulin G; C3, complement 3; C4, complement 4; PT, prothrombin time; INR, international normalized ratio; APTT, activated partial thromboplastin time; TT, thrombin time.

25(OH)D was demonstrated to be a significant predictor for MAKE risk in univariate Cox regression analysis (Supplementary Tables S2, S3). However, it was no longer significant when adjusted for all the independent factors in univariate Cox regression analysis (model 2, Supplementary Table S2). Subsequently, to substantiate the value of 25(OH)D, TWA serum 25(OH)D was calculated, and further analysis was performed. A total of 430 patients received serum 25(OH)D measurements from 2–9 times during follow-up. TWA 25(OH)D showed an AUC of 0.800 for differentiating MAKE from non-MAKE, with a cut-off value of 44.8 nmol/L (sensitivity = 68.9%, specificity = 85.1%, Figure 4A). And pairwise comparison of ROC curves found that TWA 25(OH)D performed similarly to eGFR or 24-h urinary protein (UP) in discriminating MAKE events (AUC: 0.800 vs. 0.850, *p* = 0.226; 0.800 vs. 0.740, *p* = 0.186, respectively). According to the optimal cut-off value for the risk of MAKE, patients were divided into two groups (Table 2). Compared with the higher TWA 25(OH)D group (>the cut-off value 44.8 nmol/L), patients with a lower TWA 25(OH)D (≤44.8 nmol/L) were younger, had higher levels of 24 h proteinuria, more severe Hass ‘grade, along with a lower level of serum albumin, hemoglobin, and IgG (*p* < 0.05 for each, Table 2). More importantly, the incidence of MAKE in the low-TWA 25(OH)D group was higher than that in the high-TWA 25(OH)D group (25% vs. 2.6%, *p* < 0.001, Table 2). Additionally, in the Kaplan–Meier survival analysis (Figure 4B), the cumulative incidence of MAKE was significantly higher in the lower TWA 25(OH)D group [vs. higher TWA 25(OH)D, *p* < 0.0001]. On further analyses using Spearman correlation analyses (Figure 4C), there were positive correlations between TWA 25(OH)D levels and age (*r* = 0.228; *p* < 0.001), hemoglobin (*r* = 0.230; *p* < 0.001), serum albumin (*r* = 0.261; *p* < 0.001), IgG (*r* = 0.247; *p* = 0.001), and baseline serum 25(OH)D (*r* = 0.617; *p* < 0.001) while there were negative correlations between TWA 25(OH)D levels and 24 h urinary protein (*r* = −0.242; *p* < 0.001), fibrinogen (*r* = −0.131; *p* = 0.02) and D-dimer (*r* = −0.146; *p* = 0.006) in patients with IgAN.

**Table 2:** Baseline clinicopathological characteristics stratified by different TWA of 25(OH) D level.

Parameter	Total (*n* = 430)	Low-TWA of 25(OH)D group	High-TWA of 25(OH)D group	*p*-value
		(<44.8 nmol/L, *n* = 159)	(>44.8 nmol/L, *n* = 271)	
Age (years)	39 (29, 49)	35 (27, 45.5)	41 (30, 50)	<0.001
	39 ± 12	43 ± 12	39 ± 13	
Sex (male/female)	186/244	62/97	124/147	0.206
MAKE, No. (%)	47 (11)	40 (25)	7 (2.6)	<0.001
Death, No. (%)	1 (0.2)	1 (0.6)	0 (0)	0.37
New kidney replacement therapy, No. (%)	28 (6.5)	24 (15)	4 (1.5)	<0.001
D-Scr, No. (%)	18 (4.2)	15 (9.4)	3 (1.1)	<0.001
Comorbid disease				
CKD stage (1/2/3a/3b/4)	254/87/39/31/19	89/28/15/12/15	165/59/24/19/4	0.003
Hypertension (%)	142 (33)	51 (32)	91 (34)	0.831
Cardiovascular diseases (%)	4 (0.93)	2 (1.2)	2 (0.7)	0.629
Diabetic mellitus (%)	17 (4.0)	5 (3.1)	12 (4.4)	0.687
Clinical parameter				
BMI (kg/m^2^)	23.6 (21.1, 26.1)	23.5 (20.4, 26.6)	23.7 (21.3, 26.1)	0.31
	23.2 ± 5.4	24.7 ± 3.7	23.0 ± 5.6	
SBP (mm Hg)	128 (117, 138)	128 (117, 139)	126 (118, 138)	0.481
	133 ± 75	170 ± 21	128 ± 16	
DBP (mm Hg)	82 (75, 90)	80 (75, 90)	83 (75, 90)	0.852
	82 ± 12	88 ± 13	82 ± 12	
Laboratory parameter				
Urinary protein (mg/day)	950 (354, 1,957)	1,246 (428, 2,740)	786 (330, 1,675)	0.001
	1,687 ± 2,261	2,633 ± 1,775	1,570 ± 2,289	
eGFR (mL/min/1.73 m^2^)	97 (68, 113)	96 (58, 118)	98 (70, 110)	0.833
	89 ± 30	52 ± 28	93 ± 27	
BUN (mmol/L)	5.4 (4.3, 6.7)	5.5 (4.2, 7.1)	5.4 (4.3, 6.7)	0.573
	6.0 ± 2.9	9.3 ± 5.2	5.6 ± 2.1	
Scr (μmol/L)	76.1 (60.8, 102.4)	74.4 (59.7, 116.7)	77.1 (61.1, 97.5)	0.437
	92.6 ± 51.6	164.4 ± 83.3	83.8 ± 38.0	
Uric acid (μmol/L)	353 (287, 436)	355 (279, 444)	349 (289, 428)	0.725
	365 ± 105	431 ± 109	357 ± 102	
Serum albumin (g/L)	38.6 (34.9, 42.0)	37.4 (33.8, 40.3)	39.3 (36.2, 42.7)	< 0.001
	37.9 ± 6.8	34.6 ± 6.6	38.4 ± 6.7	
Serum 25(OH)D (nmol/L)	36.6 (24.6, 48.8)	26.2 (16.5, 35.7)	43.1 (31.1, 56.3)	< 0.001
	38.4 ± 19.1	29.7 ± 14.1	39.5 ± 19.4	
TWA 25(OH)D (nmol/L)	51.3 (38.1, 65.1)	34.4 (27.7, 39.7)	61.5 (52.9, 73.3)	< 0.001
	52.8 ± 20.2	35.4 ± 20.5	54.9 ± 19.2	
FBG (mmol/L)	4.7 (4.3, 5.2)	4.7 (4.3, 5.1)	4.8 (4.4, 5.2)	0.286
	4.8 ± 0.8	4.9 ± 1.1	4.8 ± 0.8	
TG (mmol/L)	1.21 (0.88, 1.82)	1.17 (0.84, 1.87)	1.26 (0.89, 1.77)	0.935
	1.59 ± 1.34	2.33 ± 2.45	1.50 ± 1.10	
TC (mmol/L)	4.61 (4.01, 5.48)	4.57 (3.91, 5.54)	4.62 (4.10, 5.46)	0.472
	4.95 ± 1.65	4.92 ± 1.58	4.95 ± 1.66	
LDL-C (mmol/L)	3.04 (2.56, 3.75)	3.01 (2.46, 3.86)	3.05 (2.61, 3.70)	0.554
	3.26 ± 1.18	3.25 ± 1.14	3.26 ± 1.19	
HDL-C (mmol/L)	1.14 (0.93, 1.37)	1.1 (0.92, 1.30)	1.16 (0.94, 1.38)	0.169
	1.19 ± 0.37	1.06 ± 0.32	1.20 ± 0.37	
Hemoglobin (g/L)	131 (117, 143)	128 (113, 140)	133 (120, 145)	< 0.001
	130 ± 20	119 ± 22	132 ± 19	
WBC (×10^9^/L)	6.5 (5.4, 7.6)	6.6 (5.7, 7.7)	6.4 (5.3, 7.6)	0.254
	6.7 ± 1.9	6.8 ± 1.6	6.7 ± 1.9	
Neutrophil (×10^9^/L)	4.0 (3.2, 4.9)	4.1 (3.2, 5.0)	3.9 (3.2, 4.8)	0.435
	4.4 ± 3.2	4.5 ± 1.5	4.4 ± 3.4	
Neutrophil (%)	61.9 (56.3, 67.6)	61.8 (55.6, 67.6)	62.2 (56.9, 67.5)	0.927
	62.3 ± 9.0	65. 0 ± 10.3	61.9 ± 8.8	
Lymphocyte (×10^9^/L)	1.8 (1.5, 2.3)	1.9 (1.5, 2.3)	1.8 (1.5, 2.2)	0.283
	1.9 ± 0.6	1.7 ± 0.6	1.9 ± 0.6	
Lymphocyte (%)	28.8 (23.6,33.6)	28.4(23.6,33.8)	29.2(23.9,33.7)	0.971
	28.8 ± 7.8	26.0 ± 8.8	29.2 ± 7.7	
Monocyte (×10^9^/L)	0.39 (0.30, 0.49)	0.40 (0.31, 0.50)	0.38 (0.30, 0.49)	0.715
	0.41 ± 0.16	0.39 ± 0.13	0.42 ± 0.16	
Monocyte (%)	6.0 (4.9, 7.2)	5.8 (4.9, 7.5)	6.0 (5.0, 7.1)	0.517
	6.3 ± 2.1	5.8 ± 1.9	6.3 ± 2.1	
Platelet (×10^9^/L)	215 (179, 260)	215 (181, 263)	214 (179, 259)	0.841
	221 ± 64	206 ± 81	223 ± 62	
PT (s)	11.8 (11.3, 12.3)	11.9 (11.3, 12.4)	11.7 (11.3, 12.2)	0.346
	11.8 ± 0.7	11.8 ± 0.7	11.8 ± 0.8	
INR	1.03 (0.98, 1.06)	1.03 (0.98, 1.07)	1.02 (0.98, 1.06)	0.381
	1.03 ± 0.06	1.03 ± 0.06	1.03 ± 0.07	
APTT (s)	27.8 (25.9, 29.6)	27.8 (26.1, 29.9)	27.6 (25.7, 29.5)	0.376
	27.9 ± 3.6	27.8 ± 4.4	27.9 ± 3.5	
TT (s)	18.0 (17.4, 18.7)	17.9 (17.3, 18.7)	18.1 (17.5, 18.7)	0.152
	18.1 ± 1.4	18.2 ± 1.4	18.1 ± 1.4	
D-dimer (mg/L)	0.27 (0.17, 0.46)	0.29 (0.18, 0.50)	0.25 (0.16, 0.44)	0.059
	0.71 ± 4.89	0.83 ± 2.04	0.70 ± 5.13	
Fibrinogen (g/L)	2.82 (2.38, 3.44)	2.98 (2.48, 3.52)	2.76 (2.37, 3.37)	0.058
	3.02 ± 1.14	3.34 ± 1.09	2.98 ± 1.14	
IgA (g/L)	3.2 (2.5, 4.4)	3.2 (2.4, 4.6)	3.2 (2.6, 4.3)	0.683
	3.1 ± 1.1	3.2 ± 1.0	3.1 ± 1.2	
IgG (g/L)	11.6 (9.6, 15.0)	10.9 (9.3, 14.3)	12.1 (9.7, 15.2)	0.027
	11.3 ± 3.3	10.9 ± 3.4	11.3 ± 3.2	
IgM (g/L)	1.00 (0.72, 1.40)	1.00 (0.64, 1.36)	1.00 (0.72, 1.42)	0.551
	1.14 ± 0.61	1.13 ± 0.72	1.14 ± 0.60	
C3 (g/L)	1.06 (0.94, 1.27)	1.06 (0.91, 1.31)	1.08 (0.97, 1.26)	0.613
	15.49 ± 35.61	16.31 ± 37.17	15.38 ± 35.44	
C4 (g/L)	0.25 (0.20, 0.32)	0.25 (0.20, 0.33)	0.25 (0.20, 0.31)	0.724
	3.82 ± 9.2	4.52 ± 10.77	3.72 ± 8.99	
RBP (mg/L)	49.6 (38.0, 62.2)	50.3 (36.0, 63.9)	49.1 (39.0, 60.7)	0.825
	53.8 ± 22.4	67.0 ± 24.6	52.1 ± 21.6	
Pathological parameter				
Lee (1/2/3/4/5)	16/132/214/58/10	7/50/69/29/4	9/82/145/29/6	0.144
Hass (1/2/3/4/5)	43/200/124/50/13	19/60/48/27/5	24/140/76/23/8	0.018
M (0/1)	320/110	123/36	123/36	0.339
E (0/1)	422/8	157/2	265/6	0.716
S (0/1)	79/351	29/130	50/221	1
T (0/1/2)	368/48/14	136/19/4	232/29/10	0.754
C (0/1/2)	224/142/64	83/55/21	141/87/43	0.717
Medications				
Vitamin D supplementation (%)	340 (79)	29 (62)	311 (81)	0.004
Dosage of vitamin D supplementation (%)				0.006
0–400 IU/d	126 (29)	23 (49)	103 (27)	
400–1,000 IU/day	159 (37)	14 (30)	145 (38)	
>1,000 IU/day	145 (34)	10 (21)	135 (35)	
RAAS inhibitor (%)	116 (27)	49 (31)	67 (25)	0.207
Steroids and/or immunosuppressants (%)	175 (41)	73 (46)	102 (38)	0.113

BMI, body mass index; SBP, systolic blood pressure; DBP, diastolic blood pressure; eGFR, estimated glomerular filtration rate; BUN, blood urea nitrogen; Scr, serum creatine; 25(OH)D, 25-hydroxy vitamin D; FBG, fasting blood glucose; HbAc1, glycosylated hemoglobin; TG, triglyceride; TC, total cholesterol; LDL-C, low-density lipoprotein cholesterol; HDL-C, high-density lipoprotein cholesterol; WBC, white blood cell; PT, prothrombin time; INR, international normalized ratio; APTT, activated partial thromboplastin time; TT, thrombin time; URBC, urinary red blood cell; IgA, immunoglobulin A; IgG, immunoglobulin G; C3, complement 3; C4, complement 4; RBP, retinol binding protein; RAAS, renin–angiotensin–aldosterone system; MAKE, major adverse kidney events.

Data were presented as the mean ± standard deviation (SD), the median with interquartile range or counts and percentages. A two-tailed *p* < 0.05 was considered statistically significant.

### 3.4 Associations of TWA 25(OH)D with the risk of MAKE

Accordingly, multivariable-adjusted restricted cubic spline analyses indicated a significant inverse linear association between TWA 25(OH)D and MAKE (Figure 4D). To further evaluate the modification effects of subgroups on the relationship between TWA 25(OH)D and MAKE, we performed subgroup analysis in subgroups stratified by sex (male or female), age (≤60 or >60 years old), serum albumin (≤30 or >30 g/L), SBP (≤140 or >140 mm Hg), BMI (≤25 or >25 kg/m^2^), use of RAAS inhibitor (yes or no), steroids and/or immunosuppressants (yes or no) and VD supplementation (0–400, 400–1,000, or >1,000 IU/day). The lower TWA 25(OH)D remained correlated with a higher risk of MAKE (fully adjusted HR 0.962, 95% CI: 0.945–0.979, *p* < 0.001, Figure 4E). Furthermore, *p*-values for interactions were >0.05 for the subgroups by sex, age, serum albumin, SBP, BMI, use of RAAS inhibitor, steroids, and/or immunosuppressants, and VD supplementation, suggesting that the increased risk of MAKE associated with low-TWA of 25(OH)D was evident regardless of these factors.

### 3.5 Construction and internal validation of the prediction model

**Figure 5:**
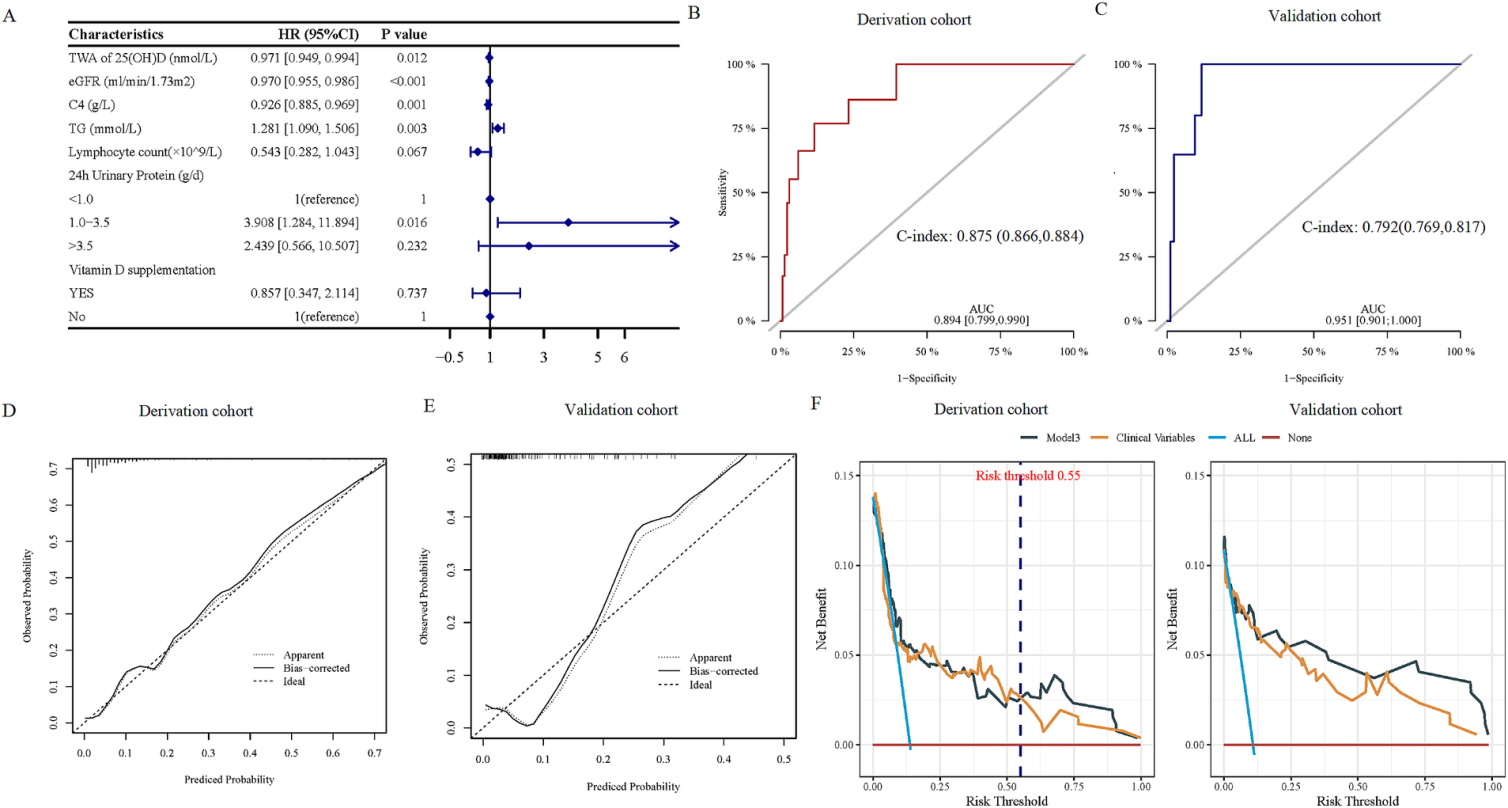
The prediction performance of the TWA 25(OH)D-based model. **(A)** Forest plots of the predictors selected by multivariate Cox analysis. **(B,C)** ROC curves for the diagnostic performance of the final Cox model for discriminating MAKE in derivation and validation cohorts, respectively. **(D,E)** Calibration curves of the final Cox model for predicting MAKE in derivation and validation cohorts, respectively. **(F)** The DCA illustrated net benefits using the two methods (TWA 25(OH)D* and clinical model) to predict MAKE in derivation (left) and validation (right) cohorts. TWA 25(OH)D* included TWA 25(OH)D and clinical factors in the clinical model. Clinical model included eGFR, 24 h UP, TG, lymphocyte count, and serum C4. MAKE, major adverse kidney events; TWA, time-weighted average; 25(OH)D, 25-hydroxy vitamin D; eGFR, estimated glomerular filtration rate; C4, complement 4; TG, triglyceride; DCA, decision curve analysis.

Next, the dataset was divided into derivation and validation cohorts using a 6:4 ratio. Details of the derivation and internal validation cohorts’ characteristics were provided in Supplementary Tables S4, S5, respectively. The higher TWA 25(OH)D was associated with a decreased risk for MAKE in unadjusted models in derivation cohorts (HR: 0.965, 95% CI: 0.946–0.984, *p* < 0.001, model 1 in Table 3). The results remained statistically significant in multivariate analysis after adjusting for all significant risk factors in univariate Cox regression (HR: 0.970, 95% CI: 0.946–0.995, *p* = 0.017, model 2 in Table 3). Independent variables were further selected by a backward stepwise multivariate Cox regression method with a *p*-value threshold of 0.05 (Figure 5A). When those variables were incorporated into the final model, lower TWA 25(OH)D remained an independent risk factor for MAKE incidence with an HR of 0.971 (95% CI: 0.949–0.994, *p* = 0.012, model 3 in Table 3). Moreover, in the reclassification analyses, adding important clinical and demographic factors (models 2 and 3 in Table 3) resulted in 60.7% (95% CI: 40.2–78.6) and 54.0% (95% CI: 37.9–74.1) improvement in the net reclassification compared with model 1 in Table 3 respectively. However, reclassification did not improve in model 3 compared to model 2 in Table 3 (−13.8, 95% CI: −26.3–3.30).

**Table 3:** Effect of TWA of 25(OH)D on MAKE.

Variable	TWA of 25(OH)D	*p*-value	AUC	NRI% (95% CI)	
Model 1 HR (95% CI)	0.965 (0.946, 0.984)	**<0.001**	0.723 (0.620, 0.826)	Reference	–
Model 2 HR (95% CI)	0.970 (0.946, 0.995)	**0.017**	0.874 (0.806, 0.943)	0.607 (0.402, 0.786)	Reference
Model 3 HR (95% CI)	0.971 (0.949, 0.994)	**0.012**	0.879 (0.810, 0.947)	0.540 (0.379, 0.741)	**−**0.138 (−0.263, 0.033)

Hazard ratios (HR) and 95% confidence intervals were derived from Cox proportional hazards regression models.

Model 1: Unadjusted.

Model 2: Model 1 plus variables with univariate Cox regression *p*-value < 0.05: sex, hypertension, hemoglobin, TG, lymphocyte, uric acid, eGFR, serum IgA, serum IgM, serum IgG, serum C3, serum C4, 24 h urinary protein, TT, vitamin D supplementation, and use of immunosuppression.

Model 3: Backward stepwise regression for model 2 plus vitamin D supplementation. Adjust variables include TG, lymphocyte, eGFR, 24 h urinary protein, serum C4, and vitamin D supplementation.

TG, triglyceride; HDL-C, high-density lipoprotein cholesterol; eGFR, estimated glomerular filtration rate; TT, thrombin time; NRI, net reclassification improvement. A two-tailed *p* < 0.05 was considered statistically significant.

Bold value: *p* < 0.05.

Consistently, the final model’s discrimination was robust, with C-statistics of 0.875 (95% CI: 0.866–0.884) in the derivation dataset and 0.792 (95% CI: 0.769–0.817) in the validation dataset (Figures 5B,C). In addition, calibration curves showed that the risk of MAKE predicted by the model was consistent with the actual probabilities in the derivation (Figure 5D) and the validation cohort (Figure 5E).

The net benefits using decision curve analysis of the derivation and the validation cohort of the TWA 25(OH)D-based model (model 3 in Table 3) [TWA of 25(OH)D*], clinical variables (TG, lymphocyte count, eGFR, 24 h urinary protein, serum C4, and VD supplementation) (clinical model), were plotted and compared. The decision curve shows that, if the risk threshold for a patient is >55%, using TWA 25(OH)D and clinical variables for predicting MAKE provides more benefits than relying solely on clinical indicators in the derivation cohort (Figure 5F). In addition, clinical decision-making based on the TWA 25(OH)D-constructed model was more beneficial than using clinical indicators alone for predicting the risk of MAKE in the validation cohort (Figure 5F).

### 3.6 Integrative analysis of key genes recognition of VD targets in IgAN

**Figure 6:**
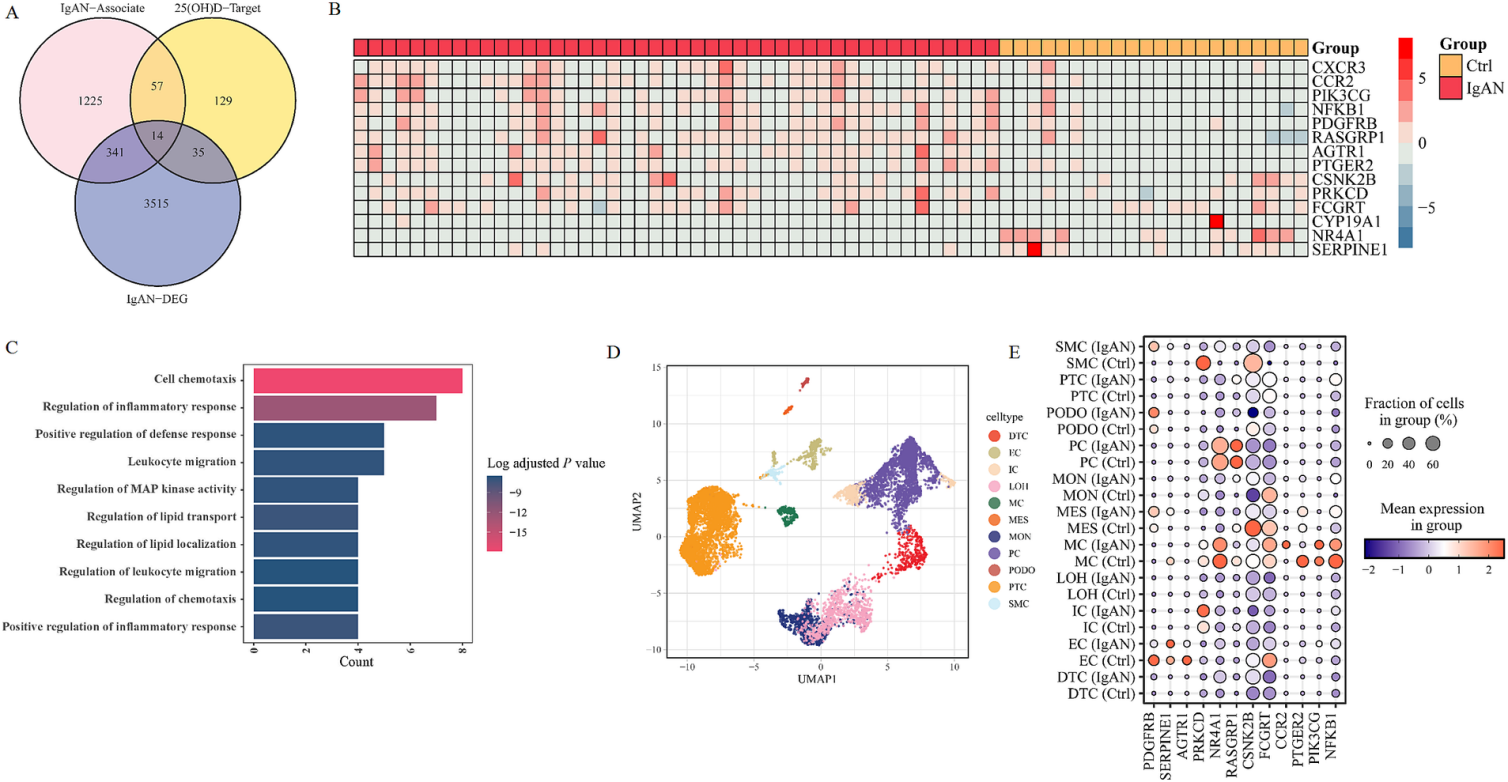
Transcriptomics analysis of IgAN-associated genes and 25(OH)D-targeted genes. **(A)** Venn diagram of IgAN-related genes, the DEGs in tissue samples of IgAN, and 25(OH)D target genes. **(B)** The heat maps of key target genes’ expression in IgAN patients. **(C)** The GO enrichment analysis of key targets. **(D)** Integrated scRNA-seq dataset of IgAN and control samples. **(E)** The heatmap of key target genes’ expressions in different cell types and groups. IgAN, IgA nephropathy; 25(OH)D, 25-hydroxy vitamin D; GO, gene ontology; DEG, differentially expressed gene; PTC, proximal tubule cells; LOH, loop of Henle cells; PC, principal cells; IC, intercalated cells; DTC, distal tubule cells; EC, endothelial cells; PODO, podocytes; MES, mesangial cell; SMC, smooth muscle cells; MC, macrophages; MON, monocytes; AGTR1, angiotensin II receptor type 1; CCR2, C–C motif chemokine receptor 2; CSNK2B, casein kinase 2 beta; FCGRT, Fc gamma receptor and transporter; NFKB1, nuclear factor kappa B subunit 1; NR4A1, nuclear receptor subfamily 4 group a member 1; PDGFRB, platelet derived growth factor receptor beta; PIK3CG, phosphatidylinositol-4,5-bisphosphate 3-kinase catalytic subunit gamma; PRKCD, protein kinase C delta; PTGER2, prostaglandin E receptor 2; RASGRP1, RAS guanyl releasing protein 1; SERPINE1, serpin family E member 1.

Subsequently, the therapeutic mechanism of VD treatments on IgAN was explored to decipher the potential mechanism of VD-disease connections and processes. First, IgAN-related genes were identified from four databases: 1,423 genes from the GeneCards database, 456 genes from the DisGeNET database, 402 genes from the OMIM database, and 12 genes from the DrugBank database. A final of 1,637 common IgAN targets were obtained. Furthermore, datasets GSE175759 were downloaded from the GEO database, and a total of 68 tissue samples (22 controls and 46 IgAN) containing 35,867 genes were merged to screen differential expression genes (DEGs). In contrast, the analysis of 25(OH)D and the identification of its potential targets were investigated. Molecular formula, molecular weight, 2D structure, and 3D structures of 25(OH)D were collected from the PubChem database (Supplementary Table S6). Then, 235 potential targets were identified by combining and removing duplicate ones from the SwissTargetPrediction database, the DrugBank database, and the SuperPred database. Next, the VD targets were intersected with the IgAN-associated genes to obtain the key genes, and a Venn diagram was plotted (Figure 6A). Finally, 14 DEGs of the 71 key genes involved in VD treatment were received, including eight upregulated and six downregulated genes (Figure 6B). Concomitantly, GO enrichment analysis showed that cell chemotaxis, regulation of inflammatory response and defense response, leukocyte migration, regulation of MAPK activity, etc. were identified as the significantly relevant signal pathways of the common targets (Figures 6C).

### 3.7 scRNA-seq and molecular docking analysis of targets

We cataloged kidney cell types of 14 key VD-targeted genes in an unbiased manner using single-cell RNA sequencing. After data pre-processing and stringent quality control, 11 clusters were identified, including proximal convoluted tubule cells (PTC), a loop of Henle (LOH) cells, distal tubule cells (DTC), principal cells (PC), intercalated cells (IC), podocytes (PODO), endothelial cells (EC), mesangial cells (MES), smooth muscle cells (SMC), macrophages (MC) and monocytes (MON) from scRNA-seq GSE171314 datasets (Figure 6D). We analyzed the expression of 14 key genes in different cell types (Figure 6E). The results showed that, compared with the control group, the nuclear factor kappa B subunit 1(NFKB1) and nuclear receptor subfamily 4, group A, member 1 (NR4A1) genes were upregulated in PTC cells, whereas the prostaglandin E receptor 2 (PTGER2) gene was downregulated in MC cells. The comprehensive and detailed distribution of the 14 common targeted genes of VD in different kidney cells was displayed in Supplementary Table S6.

**Figure 7:**
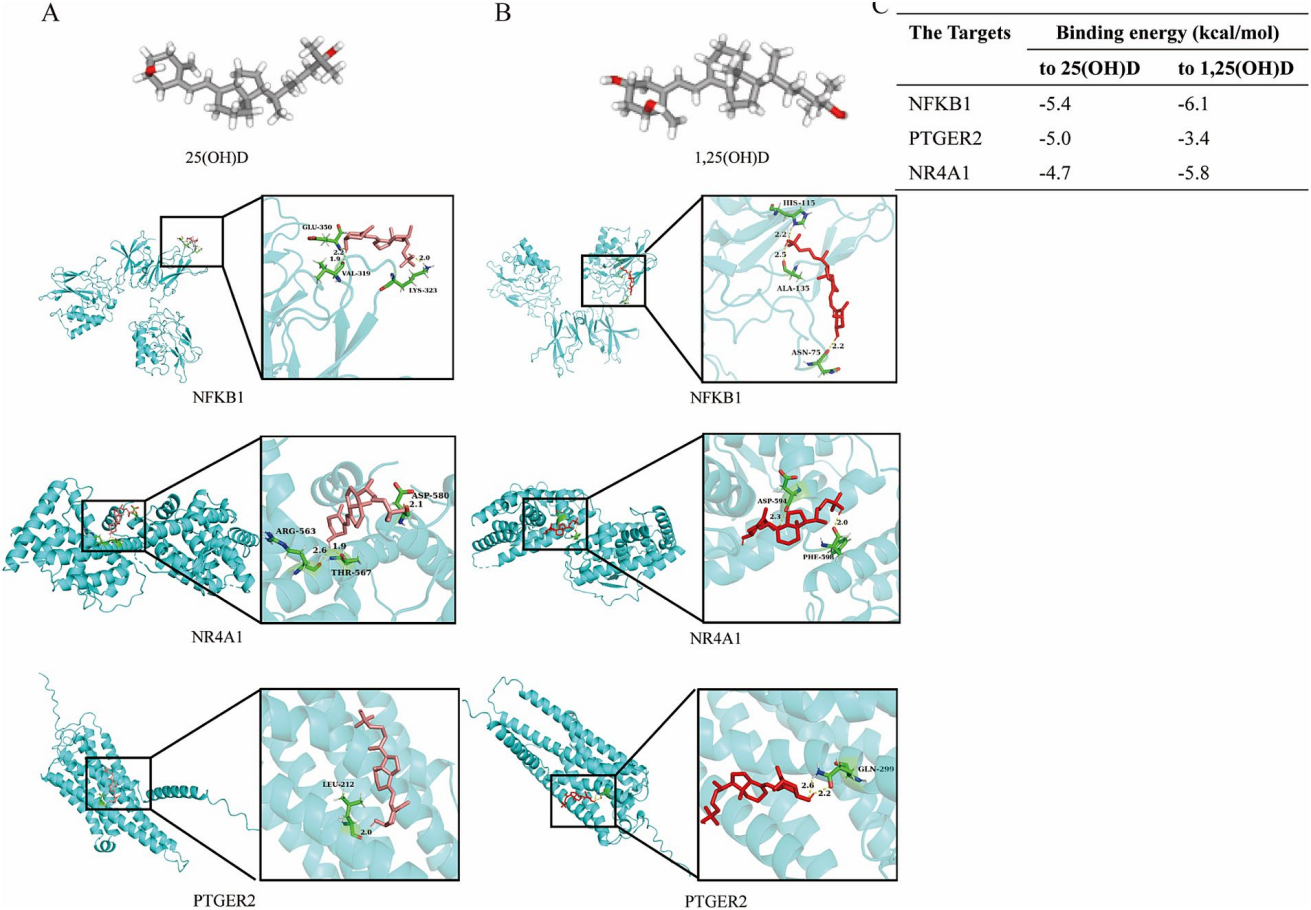
Molecular docking simulation diagram and affinity analysis. Three-dimensional (3D) structure of 25(OH)D and its molecular docking **(A)**, as well as 1,25(OH)D and its molecular docking **(B)** with the key target molecules. **(C)** Affinity of the key targets binding to 25(OH)D and 1,25(OH)D. The 25(OH)D or 1,25(OH)D molecules are pink stick models, and the protein molecules are blue cartoon models. The protein molecules at the docking site are represented as green stick models. Yellow dotted lines indicate the connected hydrogen bonds. 25(OH)D 25-hydroxy vitamin D; 1,25(OH)D 1,25 dihydroxy vitamin; NFKB1, nuclear factor kappa B subunit 1; NR4A1, nuclear receptor subfamily 4 group a member 1; PTGER2, prostaglandin E receptor 2.

Based on the data above, the 14 core target genes were individually imported into the Autodock 1.5.6 software to dock with the 25(OH)D. Affinity was the score used by the software to indicate a more substantial binding activity. Fc gamma receptor and transporter (FCGRT) and cytochrome P450 family 19 subfamily A member 1 (CYP19A1) show the highest affinity (Supplementary Table 7). The docking diagrams of 25(OH)D and genes were drawn using PyMOL software (Figure 7A; Supplementary Figure 2). In addition, we found that hydrogen bonds formed between molecules and are represented by yellow dotted lines in the diagrams. Combined with the results of scRNA-seq analysis, NFKB1, NR4A1, and PTGER2 were further docked with 1,25(OH)D (Figure 7B). Our results show that the affinities of 1,25(OH)D with NFKB1 and NR4A1 were significantly higher than those of 25(OH)D, while lower with PTGER2 (Figure 7C). A molecular docking simulation diagram of 1,25(OH)D with the other 11 key target molecules is presented in Supplementary Figure S3. Together, these results suggest that the regulation of NFKB1 and NR4A1 expressions in PTC cells may principally contribute to the potential therapeutic effects of VD supplementation in IgAN.

## 4 Discussion

The findings from this study suggest that lower serum 25(OH)D levels at baseline and follow-up are significantly associated with an increased risk of MAKE in IgAN. Moreover, total TWA 25(OH)D demonstrated robust predictive discrimination and calibration performance for the risk stratification of MAKE, with an optimal predictive cut-off value of 44.8 nmol/L. This finding underscores the critical role of VD status in modulating the risk of kidney disease progression. The results indicate a significant linear association between total TWA 25(OH)D levels and the risk of MAKE, regardless of sex, age, serum albumin, SBP, BMI, use of RAAS inhibitors, steroids, and/or immunosuppressants, and VD supplementation. Furthermore, the observed correlation between lower TWA 25(OH)D and increased MAKE risk was not limited to clinical parameters. Still, it was evident in a more nuanced reclassification of patients according to their VD status. Notably, incorporating total TWA 25(OH)D-based models into routine clinical practice has been shown to enhance overall findings, verify consistency with prior studies, and confirm the superiority of this approach over traditional risk stratification methods. Our results underscored that the long-term maintenance of optimal VD levels, even from early life stages, is associated with reduced future risk of kidney progression in IgAN. Furthermore, an integrated analysis of genomic, single-cell RNA sequencing, and molecular docking studies provided novel insights into the potential mechanisms underlying VD treatment on IgAN, revealing fresh perspectives for delaying disease progression and strengthening therapeutic theories.

Here, we reported 74% [25(OH)D < 50 nmol/L], 25% [25(OH)D < 25 nmol/L], or 33% [25(OH)D < 30 nmol/L] of patients with IgAN had VD deficiency according to standards of international guidelines, which suggested that individuals with IgAN were at high risk of inadequate VD levels. Numerous data have indicated that 25(OH)D deficiency/insufficiency is common in CKD patients, and its prevalence increases with increasing CKD severity (8). One multicenter study from several US regions indicated that 25(OH)D deficiency was prevalent in 71 and 83% of patients with moderate and severe CKD not on dialysis (23). A consistent pattern was observed across all stages of CKD in IgAN, where baseline and total 25(OH)D levels were decreased. Several factors have been proposed to contribute to this suboptimal status, including reduced exposure to sunlight, impaired endogenous synthesis of VD in response to biologically active ultraviolet radiation (UVB) irradiation, and lower dietary intake of VD sources (24). Additionally, a recent study suggests that differences in 25(OH)D clearance by kidney function and ethnicity may contribute to VD metabolism in patients with CKD (25). Furthermore, a vast body of evidence suggested an independent link between VD deficiency [25(OH)D < 50 nmol/L] and mortality in CKD for its extra-skeletal effects (26–28). The most extensive cohort analysis among people with non-dialysis CKD, the Third National Health and Nutrition Examination Survey (NHANES-III) showed that a lower 25(OH)D concentration (<37.5 nmol/L, lowest tertile) was significantly associated with all-cause mortality (hazard ratio [HR] 1.56; 95% CI: 1.12, 2.18) compared with the highest tertile (>75 nmol/L) (29). A meta-analysis that included four studies of non-dialysis patients found a 14% lower all-cause mortality for each 25 nmol/L higher 25(OH)D concentration, regardless of the stage of CKD (30). Moreover, a recent non-linear Mendelian randomization analysis of UK Biobank participants supports a causal relationship between VD deficiency and mortality in the general population (31). The consistency with which these studies corroborate the notion that patients exhibiting baseline serum 25(OH)D levels below 57.2 nmol/mL not only precipitated more severe renal functional decline and a broader range of pathological manifestations but also exhibited a significantly elevated propensity for developing nephropathic disorders as evidenced by the Kaplan–Meier analysis. Thus, discerning the fundamental threshold of serum 25(OH)D values may prove to be a pivotal factor in elucidating the predilections of high-risk patients for renal complications and thereby guiding the judicious commencement of VD supplementation in individuals displaying IgAN.

Moreover, the Threshold Value Associated with TWA 25(OH)D exhibited an outstanding capacity for distinguishing between high-risk and low-risk individuals for kidney disease progression, as evidenced by its impressive discriminatory power, which manifests comparable performance to readily available routine biomarkers, such as eGFR and 24 h proteinuria. Consistently across diverse methods, the results showed that TWA 25(OH)D systematically inversely correlated with the risk of developing nephropathic complications in IgAN patients, rendering it an indispensable tool for assessing the likelihood of adverse outcomes. Notably, our analysis revealed that baseline 25(OH)D levels displayed limited predictive value for the risk of nephropathic complications in IgAN. After adjusting multiple factors, the predictive ability of baseline 25(OH)D levels for MAKE decreased significantly and did not reach statistical significance. This finding provides a plausible explanation for the divergent outcomes observed between baseline VD deficiency and renal prognosis in patients with IgAN (12, 17). Baseline VD levels are influenced by a variety of factors, including outdoor exercise, sun exposure, nutritional status, seasonal changes, and dietary habits. While the baseline status is associated with disease severity, it may not be a reliable predictor for the risk of future kidney damage. In contrast, a TWA threshold level was identified as an independent risk factor for MAKE, even after adjusting for all relevant covariates in multivariate Cox regression analyses. To subsequently evaluate the chronic trajectory of renal disease in this patient cohort, it is recommended that clinicians prioritize ongoing monitoring of VD levels to maintain optimal targets, specifically 44.8 nmol/L. The findings suggest that sustained elevation of VD concentrations may be associated with reduced subsequent risk of progressive kidney dysfunction and mortality in patients with IgAN.

Another noteworthy outcome of this study was the comprehensive integration of various omics analyses—integrated network pharmacology analysis, molecular docking, and single-cell RNA sequencing—which provided complementary perspectives on the potential mechanisms underlying the effects of VD treatment on IgAN. Previous research had established that VD therapy enhances the antiproteinuric effect of angiotensin-converting enzyme inhibitors (ACEIs)/angiotensin receptor blockers (ARBs), a recommended initial treatment approach for the control of proteinuria and slowing disease progression in IgAN (5, 32, 33). One key aspect of the Reno protective and anti-proteinuria effects of VD is its potential to modulate various biological pathways involved in kidney injury. Specifically, VD may exert anti-inflammatory, antineutrophilic, and tissue-protective effects that contribute to reduced inflammation, suppression of the RAAS, and ultimately, reduction of interstitial fibrosis, podocyte damage, and glomerulosclerosis (34). In our study, this point has also been further corroborated by multi-omics studies in IgAN. Our findings underscored the intricate relationship between VD targets and inflammation in IgAN. Specifically, we observed that VD may influence cellular processes such as chemotaxis and inflammatory response. Moreover, our results suggest that modulating the chemotaxis of Th22 cells could be a promising therapeutic strategy for mitigating inflammation in IgAN, as these cells are implicated in both the initiation and progression of the disease (35–38). However, whether VD treatment inhibits Th22 cell chemotaxis to regulate inflammatory disorders, thereby leading to an improvement in kidney function in IgAN, remains the subject of future studies.

The canonical VD activation pathway involves 25- and subsequent 1α-hydroxylation, whereby the VD metabolite 25(OH)D produced in the liver is subsequently taken up by renal proximal tubular cells and metabolized to 1,25(OH)D (39). The proximal tubule is the high-capacity resorptive powerhouse of the kidney, which is the primary source of circulating 1,25(OH)D under physiological conditions (39, 40). Our results revealed that NFKB1, NR4A1, and PTGER2 might be key candidate genes for VD treatment in IgAN. NFKB1 encodes the transcription factor nuclear factor kappa B1 (NF-κB1), which plays a crucial role in regulating the survival, activation, and differentiation of innate immune cells and inflammatory T cells, and participates in inflammation development and progression (41, 42). One study by Yuan et al. found that a combination therapy of VD and tacrolimus can effectively improve renal tissue damage in the IgAN rat model by regulating immune response via NF-κB/TLR4 pathway (43). In contrast, NR4A1, as a member of the nuclear hormone receptor superfamily, contributes to the regulation of inflammation, oxidative stress, and cell apoptosis in kidney disease, with the potential to maintain the homeostasis of lymphocytes and participate in T cell differentiation (44). In addition, in multiple sclerosis patients, 1,25(OH)D could activate repressed apoptosis via NR4A1 expression, potentially leading to better disease control by destroying autoreactive cells. In heart disease, VD can bind to the VD receptor (VDR) in the heart to regulate the activity of NR4A1 and play a protective role (45, 46). Collectively, VD treatment, 25(OH)D, and 1,25(OH)D analogs may hold great clinical promise for alleviating the processes of inflammation, immunity, and metabolism in IgAN by modulating the expression of NFKB1 and NR4A1 in PTC cells. However, the interaction between NFKB1/NR4A1 and VD was predicted solely through computational simulations. Further in-depth studies, including *in vitro* and *in vivo* experimental validation (e.g., gene knockout or overexpression), are still warranted to clarify the specific mechanistic processes involved.

Our study has several strengths. First, by providing extensive sample data and follow-up, we demonstrated that TWA 25(OH)D was an independent determinant for MAKE in IgAN, where the association was significant inverse linearity, and the magnitude of the association was consistent across subgroup analyses. Second, the findings guide clinicians toward a dynamic monitoring of their circulating levels and optimal dosing regimens of IgAN in clinical practice. Third, in addition to well-accepted routine clinical parameters, TWA 25(OH)D had high clinical value in risk stratification of kidney outcomes in IgAN, which has been verified internally and demonstrated satisfactory efficacy and significant net advantages. Our model may provide important references for individualized risk stratification and optimized treatment strategies. Fourth, the integrated analysis of genomic, scRNA-seq, and molecular docking data offers a complementary and more comprehensive understanding of VD treatment, strengthening the role of VD in IgAN.

Our findings are subject to the following limitations. First, although the sample size is relatively large and the follow-up period is long, it is a single-center retrospective cohort study with only internal validation. More prospective, multicenter, and well-designed studies with external validation are still warranted in the future. Second, potential confounding factors affecting 25(OH)D levels, such as geographies, outdoor exercise, sun exposure, nutritional status, seasonal alternation, dietary habits, and bone metabolism markers, may be better included in future research. As such, continuing in-depth research regarding the value of VD in IgAN warrants further studies aimed at patient-oriented outcomes, which include screening for VD status (circulating levels at baseline and follow-up), sunlight exposure, outdoor exercise, and dosing patterns and initiation timing of supplementation and detailed treatment schedule (daily, weekly, or monthly intervals).

## 5 Conclusion

In conclusion, our data suggest that lower serum 25(OH)D levels at baseline and follow-up were significantly associated with an increased risk of MAKE in IgAN. Moreover, total 25(OH)D (TWA) levels were an independent determinant for MAKE in IgAN, revealing a significant inverse linear association. This finding was consistent across subgroup analyses and highlights the importance of considering TWA levels as a risk factor for adverse health effects. Furthermore, our results demonstrate that the TWA-based model exhibited strong risk prediction power, allowing clinicians to identify high-risk patients and intervene early to halt disease progression. Notably, this model’s ability to predict risk is not only limited to clinical parameters but also incorporates insights from genomic, scRNA-seq, and molecular docking analyses into the underlying mechanisms of IgAN. The integrative analysis of these data suggests that VD treatment may play a crucial role in improving the prognosis for patients with IgAN. Specifically, our findings suggest that maintaining optimal 25(OH)D levels from early life may be associated with reduced future risk of kidney progression. This highlights the potential value of considering circulating 25(OH)D levels as an individualized tool for clinical decision-making for patients with IgAN. In conclusion, our study provides evidence supporting the importance of VD in IgAN. It suggests that maintaining optimal 25(OH)D levels may be a key factor in slowing the progression of future kidney disease. We recommend that clinicians pay closer attention to circulating 25(OH)D levels and consider this information when making treatment decisions for patients with IgAN.

## Data Availability

Raw data, processed data, and metadata from human single-cell dataset have been deposited in GEO with the accession number GSE171314 (https://www.ncbi.nlm.nih.gov/geo/query/acc.cgi?acc=GSE171314). Accession number of RNA-seq profiling of human kidney samples was GSE175759 (https://www.ncbi.nlm.nih.gov/geo/query/acc.cgi?acc=GSE175759). Source data of cohort will be supplied by the corresponding authors with a reasonable request.

## Footnotes

^1^ https://www.disgenet.org/

^2^ https://www.genecards.org

^3^ https://go.drugbank.com/

^4^ https://omim.org/

^5^ https://pubchem.ncbi.nlm.nih.gov/

^6^ http://swisstargetprediction.ch/

^7^ http://prediction.charite.de

^8^ https://www.uniprot.org/

^9^ http://www.ncbi.nlm.nih.gov/geo

^10^ https://bioconductor.org/packages/release/bioc/html/edgeR.html

^11^ https://CRAN.R-project.org/package=VennDiagram

^12^ https://www.rcsb.org/

^13^ https://pymol.org

^14^ http://autodock.scripps.edu/

^15^ https://www.R-project.org/

^16^ https://CRAN.R-project.org/package=rmda

^17^ https://CRAN.R-project.org/package=survminer
