# Supplementary material for: Effect of time-weighted average 25(OH)D on the occurrence of major adverse kidney events in IgA nephropathy—from a 10-year population-based cohort study
Source: Front Nutr. 2025 Aug 1;12:1576488. doi: 10.3389/fnut.2025.1576488 (PMC12353751; doi:10.3389/fnut.2025.1576488)
Supplement: Supplementary file 1 [file Data_Sheet_1.pdf]

Supplementary Figure 1. Distribution of TWA 25(OH)D levels in IgAN patients.

(A). Four categories of TWA 25(OH)D levels according to the Endocrine Society clinical practice (ESC) guidelines and the UK Scientific Advisory Committee on Nutrition (SACN). (B). Three categories of TWA 25(OH)D levels according to the Institute of Medicine (IOM). (C). Significant difference was obtained in TWA 25(OH)D levels between male and female, in different groups of 24h UP, and at different stages of CKD, respectively. **p* < 0.05, ***p* < 0.01, ****p* < 0.001, *****p* < 0.0001.

TWA time-weighted average; 25(OH)D 25-hydroxyvitamin D; 24h UP 24h urinary protein; CKD chronic kidney disease.

Supplementary Figure 2. Molecular docking simulation diagram of 25(OH)D.

3D Structure of molecular docking of 25(OH)D with the other eleven key targets molecules. The 25(OH)D molecules are pink stick models and the protein molecules are green cartoon models. The protein molecules at the docking site are represented as violet stick models. The connected hydrogen bonds are indicated by yellow dotted lines.

25(OH)D 25-hydroxy vitamin D; AGTR1, angiotensin II receptor type 1; CCR2, c-c motif chemokine receptor 2; CSNK2B, casein kinase 2 beta; FCGRT, fc gamma receptor and transporter; PDGFRB, platelet derived growth factor receptor beta; PIK3CG, phosphatidylinositol-4,5-bisphosphate 3-kinase catalytic subunit gamma; PRKCD, protein kinase c delta; RASGRP1, RAS guanyl releasing protein 1; SERPINE1, serpin family e member 1.

Supplementary Figure 3. Molecular docking simulation diagram of 1,25(OH)D.

3D Structure of molecular docking of 1,25(OH)D with the other eleven key targets molecules. The 1,25(OH)D molecules are red stick models and the protein molecules are green cartoon models. The protein molecules at the docking site are represented as violet stick models. The connected hydrogen bonds are indicated by yellow dotted

lines.

1,25(OH)D 1,25 dihydroxy vitamin D; AGTR1, angiotensin II receptor type 1; CCR2, c-c motif chemokine receptor 2; CSNK2B, casein kinase 2 beta; FCGRT, fc gamma receptor and transporter; PDGFRB, platelet derived growth factor receptor beta; PIK3CG, phosphatidylinositol-4,5-bisphosphate 3-kinase catalytic subunit gamma; PRKCD, protein kinase c delta; RASGRP1, RAS guanyl releasing protein 1; SERPINE1, serpin family e member 1.

Supplementary Figure 1

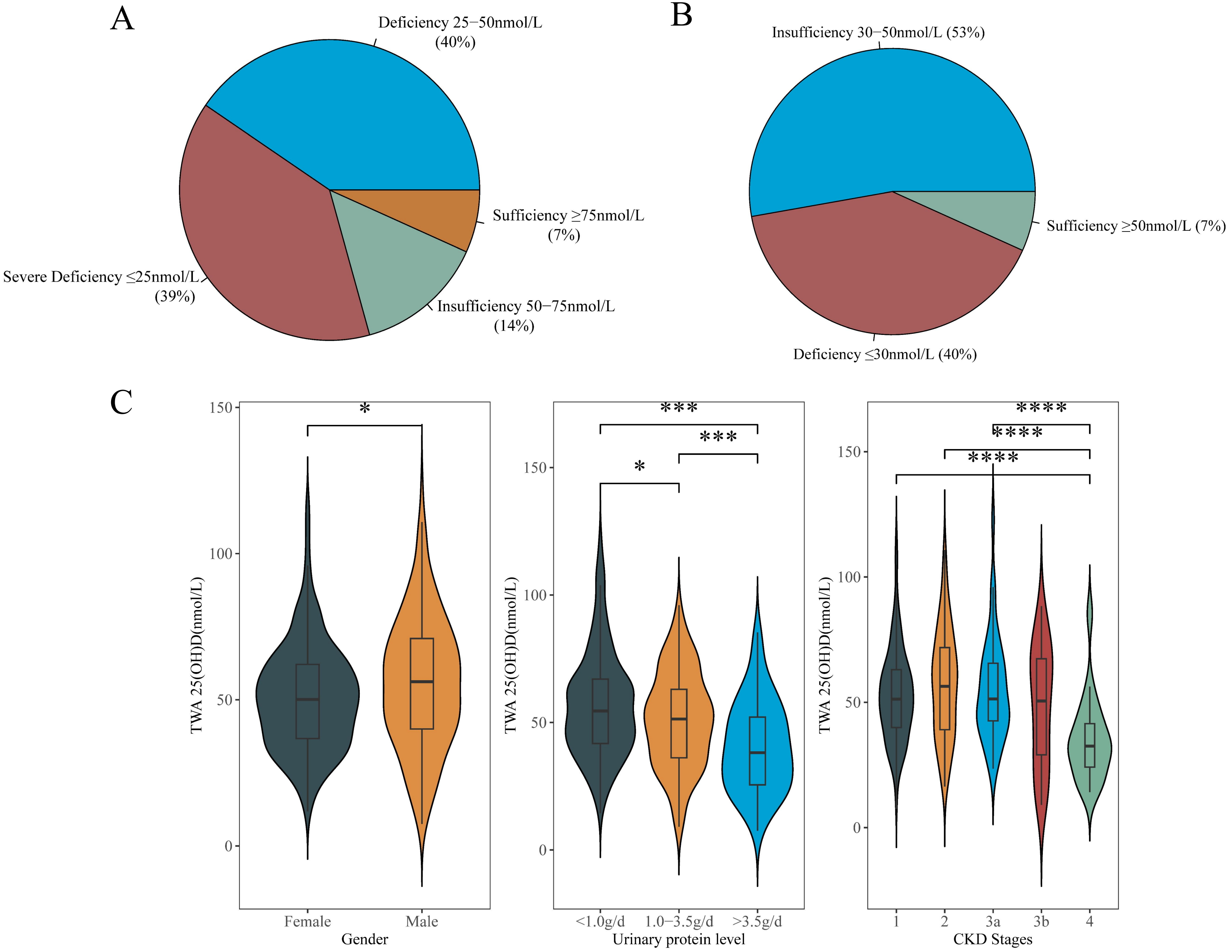

Supplementary Figure 2

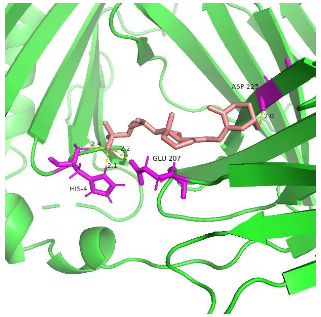

FCGRT

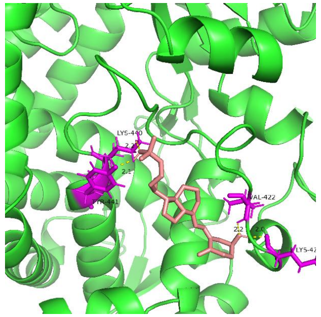

CYP19A1

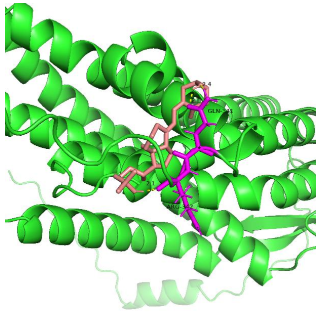

CXCR3

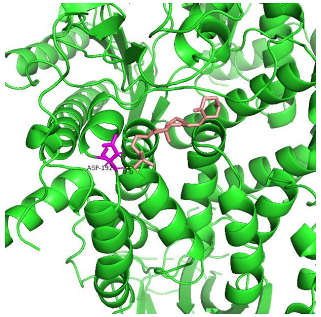

PIK3CG

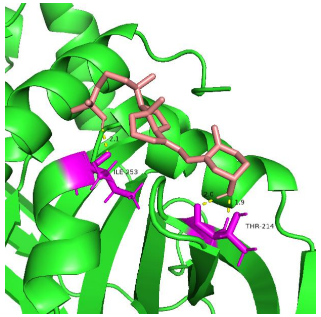

SERPINE1

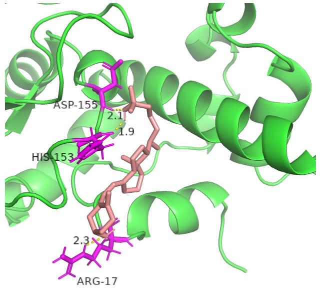

CSNK2B

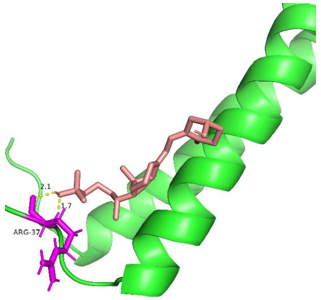

PDGFRB

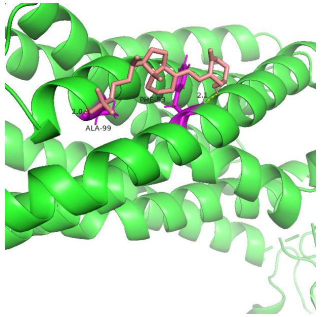

CCR2

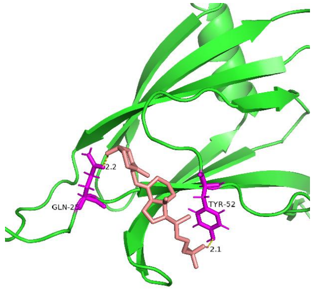

PRKCD

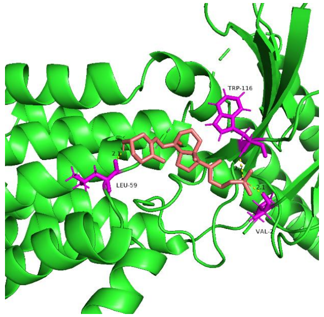

ATGR1

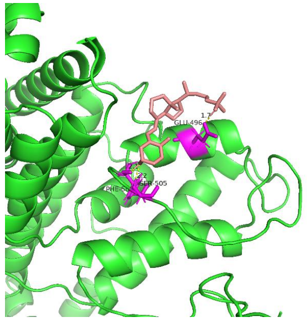

RASGRP1

Supplementary Figure 3

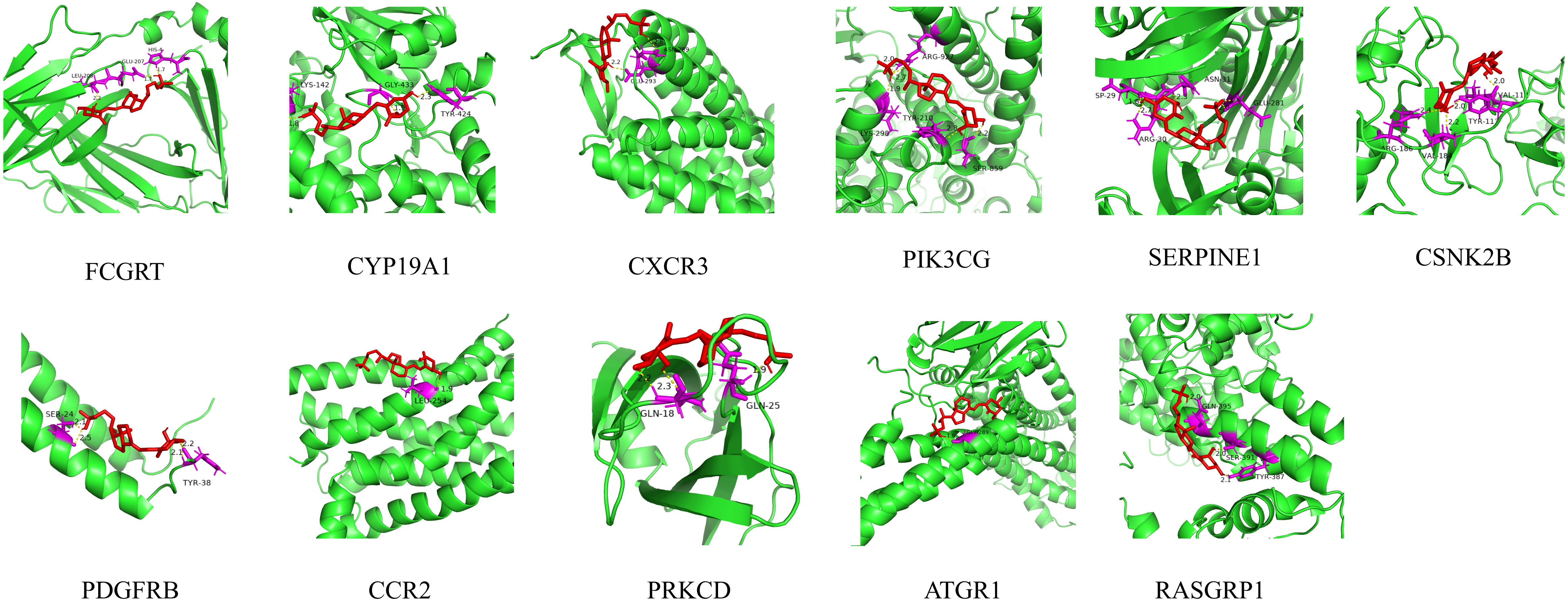

Supplementary Table 1. Baseline clinicopathological characteristics stratified by different vitamin D level.

Parameter	Low-25(OH)D group (＜57.2nmol/L, n=727)	High-25(OH)D group (≥57.2nmol/L, n=139)	*P* value
Age (years)	38 (28, 48)	43 (35, 51.5)	< 0.001
Gender (male/female)	318/409	89/50	< 0.001
MAKE, No. (%)	87 (12)	5 (3.6)	0.005
Death, No. (%)	3 (0.004)	0 (0)	1
New kidney replacement therapy, No. (%)	53 (7.3)	5 (3.6)	0.158
D-Scr, No. (%)	31 (4.3)	0 (0)	0.006
**Comorbid disease**			
CKD stage (1/2/3a/3b/4)	433/150/59/54/31	74/38/16/6/5	0.163
Hypertension (%)	232 (32)	51 (37)	0.316
Cardiovascular diseases (%)	9 (1.2)	2 (1.4)	0.693
Diabetic mellitus (%)	34 (4.6)	8 (5.8)	0.604
**Clinical parameter**			
BMI (kg/m^2)	23.6 (21.2, 26.6)	24.3 (21.8, 26.2)	0.452
SBP (mmHg)	128 (118, 140)	126 (117, 138)	0.293
DBP (mmHg)	82 (75, 90)	82 (73, 90)	0.549
**Laboratory parameter**			
Urinary protein excretion (mg/d)	944 (363, 2118)	389 (211, 833)	< 0.001
eGFR (ml/min/1.73 m^2)	98 (68, 114)	93 (67, 107)	0.086
BUN (mmol/L)	5.3 (4.3, 6.7)	5.6 (4.7, 6.8)	0.128
Scr (μmol/L)	74.8 (60.4, 104.6)	85.6 (68.6, 103.1)	0.03
Uric acid (μmol/L)	352 (286, 436)	387 (304, 450)	0.047
Serum albumin (g/L)	38.5 (34.8, 41.8)	40.5 (37.9, 44.5)	< 0.001
Serum 25(OH)D (nmol/L)	33.5 (23.9, 43.0)	66.9 (61.6, 77.6)	< 0.001
FBG (mmol/L)	4.7 (4.4, 5.2)	4.9 (4.4, 5.3)	0.019
TG (mmol/L)	1.29 (0.90, 1.92)	1.23 (0.92, 1.78)	0.745
TC (mmol/L)	4.60 (3.96, 5.50)	4.51 (3.92, 5.24)	0.128
LDL-C (mmol/L)	3.02 (2.52, 3.76)	2.87 (2.49, 3.51)	0.078
HDL-C (mmol/L)	1.12 (0.93, 1.35)	1.09 (0.96, 1.25)	0.185
Hemoglobin (g/L)	131 (118, 144)	137 (127, 147)	< 0.001
WBC(×10^9/L)	6.7 (5.6, 8.0)	6.4 (5.4, 7.2)	0.01
Neutrophil (×10^9/L)	4.1 (3.3, 5.2)	3.9 (3.3, 4.7)	0.092
Neutrophil (%)	62 (57, 68)	63 (56, 68)	0.838
Lymphocyte (×10^9/L)	1.9 (1.5, 2.3)	1.7 (1.4, 2.1)	0.032
Lymphocyte (%)	29 (23, 34)	28 (24, 33)	0.344
Monocyte (×10^9/L)	0.39 (0.31, 0.50)	0.39 (0.29, 0.49)	0.708
Monocyte (%)	5.9 (4.8, 7.1)	6.0 (5.1, 7.3)	0.201
Platelet (×10^9/L)	220 (180, 260)	199 (153, 247)	< 0.001
PT (s)	11.8 (11.4, 12.3)	11.9 (11.4, 12.5)	0.111
INR	1.03 (0.98, 1.07)	1.03 (0.99, 1.08)	0.142
APTT (s)	27.8 (25.9, 29.8)	27.9 (25.7, 29.5)	0.778

TT (s)	18.1 (17.5, 18.8)	18.3 (17.5, 18.9)	0.506
D-dimer (mg/L)	0.25 (0.16, 0.44)	0.23 (0.15, 0.37)	0.144
Fibrinogen (g/L)	2.84 (2.34, 3.47)	2.68 (2.37, 3.23)	0.143
URBC (/uL)	92 (32, 263)	84 (28, 235)	0.273
IgA (g/L)	3.1 (2.4, 4.0)	3.2 (2.6, 4.3)	0.09
IgG (g/L)	11.4 (9.4, 14.4)	12.5 (10.5, 15.1)	0.004
IgM (g/L)	1.00 (0.72, 1.44)	1.06 (0.74, 1.42)	0.473
C3 (g/L)	1.08 (0.94, 1.30)	1.06 (0.97, 1.22)	0.581
C4 (g/L)	0.26 (0.2, 0.33)	0.26 (0.22, 0.31)	0.491
RBP (mg/L)	50.3 (38.0, 64.1)	50.3 (40.9, 63.3)	0.445
**Pathological parameter**			
Lee (1/2/3/4/5)	27/221/366/89/24	5/44/77/11/2	0.466
Hass (1/2/3/4/5)	81/332/203/82/29	16/69/41/10/3	0.492
M (0/1)	544/183	105/34	0.944
E (0/1)	710/17	138/1	0.335
S (0/1)	139/588	31/108	0.454
T (0/1/2)	622/86/19	124/10/5	0.209
C (0/1/2)	399/232/96	80/40/19	0.765
**Medications**			
RAAS inhibitor (%)	183 (25)	37 (27)	0.732
Steroids and/or immunosuppressants (%)	355 (49)	67 (48)	0.965

BMI Body mass index; SBP systolic blood pressure; DBP diastolic blood pressure; eGFR estimated glomerular filtration rate; BUN blood urea nitrogen; Scr serum creatine; 25(OH)D 25-hydroxy vitamin D; FBG fasting blood glucose; HbAc1 Glycosylated Hemoglobin; TG triglyceride; TC total cholesterol; LDL-C low-density lipoprotein cholesterol; HDL-C high-density lipoprotein cholesterol; WBC white blood cell; PT prothrombin time; INR International normalized ratio; APTT activated partial thromboplastin time; TT thrombin time; URBC urinary red blood cell; IgA Immunoglobulin A; IgG Immunoglobulin G; C3 complement 3; C4 complement 4; RBP Retinol binding protein; RAAS Renin-Angiotensin-Aldosterone System; MAKE Major adverse kidney events.

Data were presented as the mean±standard, the median with interquartile range or counts and percentages. A two-tailed $p<0.05$ was considered statistically significant.

Supplementary Table 2. Effect of baseline 25(OH)D on MAKE.

Variable	baseline 25(OH)D	*P* value
Model 1 HR (95%CI)	0.986(0.975, 0.998)	0.022
Model 2 HR (95%CI)	1.003(0.988, 1.017)	0.861

Hazard ratios (HR) and 95% confidence intervals were derived from Cox proportional hazards regression models.

Model 1: unadjusted

Model 2: Model 1 plus variables with univariate cox regression p value <0.05: Age, Hypertension, Cardiovascular diseases, Hemoglobin, TG, Lymphocyte, Uric acid, eGFR, Serum IgA, Serum IgG, Serum C3, Serum C4, 24h urinary protein, and use of steroids and/or immunosuppressants.

TG triglyceride; HDL-C high-density lipoprotein cholesterol; eGFR, estimated glomerular filtration rate; TT thrombin time; NRI net reclassification improvement. A two-tailed $P<0.05$ was considered statistically significant.

Supplementary Table 3. Predictors of MAKE identified by Univariate Cox regression analysis in all patients

**Variable**	**Univariate Cox Regression**	
	**HR (95%CI)**	***P-value***
Clinical features		
Age (incremented by 1 years)	1.017 (1.001-1.032)	0.038
BMI (incremented by 1 kg/m^2)	1.062 (1.022-1.104)	0.002
SBP (incremented by 1 mmHg)	1.001 (1.000-1.003)	0.021
Hemoglobin (incremented by 1 g/L)	0.965 (0.954-0.975)	< 0.001
TG (incremented by 0.1 mmol/L)	1.142 (1.048-1.244)	0.002
BUN (incremented by 1 mmol/L)	1.136 (1.106-1.167)	< 0.001
eGFR (incremented by 1 ml/min/1.73m^2)	0.970 (0.964-0.977)	< 0.001
Scr (incremented by 1 mmol/L)	1.010 (1.008-1.012)	< 0.001
Uric acid (incremented by 1 μmol/L)	1.004 (1.002-1.005)	< 0.001
C4 (incremented by 0.01 g/L)	0.952 (0.928-0.977)	< 0.001
25(OH)D (incremented by 1 nmol/L)	0.988 (0.976-0.999)	0.036
Lymphocyte count (incremented by 0.1×10^9/L)	0.570 (0.402-0.809)	0.002
Pathological features		
S score		
0	Reference	1
1	2.534 (1.227-5.231)	0.012
T score		
0	Reference	1
1	2.139 (0.780-5.866)	0.14
2	2.066 (1.216-3.512)	0.007
C score		
0	Reference	1
1	1.065 (0.646-1.756)	0.805
2	3.069 (1.799-5.234)	< 0.001
Medications		
Use of Steroids and/or immunosuppressants (Yes vs. No)	0.447 (0.294-0.680)	< 0.001
Dosage of Vitamin D supplementation		
0-400 IU/d	Reference	1
400-1000 IU/d	1.094 (0.687-1.739)	0.706
>1000 IU/d	0.408 (0.208-0.802)	0.009

Hazard ratios (HR) and 95 % confidence intervals were derived from Cox proportional hazards regression models. BMI Body mass index; SBP systolic blood pressure; TG triglyceride; BUN blood urea nitrogen; eGFR estimated glomerular filtration rate; Scr, serum creatine; C4 complement 4; 25(OH)D serum 25-hydroxyvitamin D

Supplementary Table 4. Baseline clinicopathological characteristics stratified by different TWA 25(OH) D level in training set.

**Parameter**	**Total (n=258)**	**Low-TWA 25(OH)D group (＞44.8nmol/L，n=160)**	**High-TWA 25(OH)D group (＜44.8nmol/L，n=98)**	**P value**
Age (years)	39 (29, 49)	41 (30, 50)	36 (27, 45)	0.009
Gender (male/female)	107/151	72/88	35/63	0.181
MAKE, No. (%)	32(12)	6(3.8)	26(27)	<0.001
**Comorbid disease**				
CKD stage (1/2/3a/3b/4)	153/51/24/19/11	100/31/16/11/2	53/20/8/8/9	0.049
Hypertension (%)	90 (35)	58 (36)	32 (33)	0.65
Cardiovascular diseases (%)	3 (1.2)	2 (1.3)	1 (1.0)	1
Diabetic mellitus	10 (3.9)	8 (5.0)	2 (2.0)	0.327
**Clinical parameter**				
BMI (kg/m2)	23.4 (21.0, 25.9)	23.8 (21.1, 26.0)	22.9 (20.5, 25.8)	0.311
SBP (mmHg)	128 (117, 139)	127(117, 138)	128 (119, 139)	0.43
DBP (mmHg)	82.6 ± 12.3	82.6 ± 11.3	82.6 ± 13.9	0.989
**Laboratory parameter**				
Urinary protein(mg/d)	931 (357, 1853)	731 (338, 1627)	1168(420, 2578)	0.024
eGFR (ml/min/1.73 m^2)	99 (68, 114)	99 (74, 111)	97 (60, 118)	0.691
BUN (mmol/L)	5.3 (4.2, 6.7)	5.3 (4.3, 6.6)	5.5 (4.2, 7.2)	0.423
Scr (μmol/L)	73.1 (60.0, 104.2)	73.1 (60.9, 97.6)	72.8 (59.3, 115.5)	0.622
Uric acid (μmol/L)	349 (283, 429)	346 (287, 421)	354 (276, 442)	0.703
Serum albumin (g/L)	38.6 (35.2, 41.8)	39.1 (36.2, 42.7)	37.6 (34.4, 40.3)	0.01

Serum 25(OH)D (nmol/L)	36.1 (24.9, 48.2)	42.2 (30.8, 54.9)	27.4 (16.6, 35.2)	< 0.001
TWA of 25(OH)D (nmol/L)	51.5 (37.8, 65.3)	62.4 (53.7, 74.8)	34.4 (30.0, 40.3)	< 0.001
FBG (mmol/L)	4.8 (4.3, 5.2)	4.7 (4.3, 5.2)	4.8 (4.4, 5.1)	0.925
TG (mmol/L)	1.17 (0.86, 1.72)	1.20 (0.84, 1.71)	1.17 (0.92, 1.78)	0.546
TC (mmol/L)	4.61 (4.03, 5.48)	4.62 (4.12, 5.49)	4.53 (3.94, 5.45)	0.389
LDL-C (mmol/L)	3.01 (2.54, 3.78)	3.06 (2.60, 3.74)	2.94 (2.48, 3.81)	0.508
HDL-C (mmol/L)	1.12 (0.93, 1.38)	1.15 (0.95, 1.41)	1.10 (0.92, 1.3)	0.087
Hemoglobin(g/L)	129.8 ± 18.0	132.4 ± 17.9	125.6 ± 17.4	0.003
WBC(×10^9/L)	6.5 (5.5, 7.6)	6.4 (5.4, 7.6)	6.7 (5.7, 7.6)	0.424
Neutrophil(×10^9/L)	4.1 (3.3, 4.9)	4.0 (3.3, 4.8)	4.1 (3.2, 5.0)	0.671
Neutrophil (%)	62.5 ± 9.1	62.7 ± 9.5	62.1 ± 8.4	0.635
Lymphocyte(×10^9/L)	1.8 (1.4, 2.3)	1.8 (1.4, 2.3)	1.9 (1.5, 2.4)	0.359
Lymphocyte (%)	28.6 ± 7.9	28.6 ± 8.2	28.6 ± 7.3	0.978
Monocyte(×10^9/L)	0.39 (0.30, 0.51)	0.37 (0.30, 0.50)	0.41 (0.32, 0.51)	0.245
Monocyte (%)	5.95 (4.90, 7.27)	5.90 (4.77, 7.12)	6.05 (5.10, 7.68)	0.526
Platelet (×10^9/L)	218.5 ± 62.9	217.5 ± 60.2	220.1 ± 67.3	0.75
PT(s)	11.82 ± 0.76	11.79 ± 0.74	11.88 ± 0.79	0.356
INR	1.03 ± 0.07	1.03 ± 0.07	1.04 ± 0.07	0.256
APTT(s)	27.9 (26.0, 29.9)	28.0 (26.1, 29.7)	27.6 (26, 30.2)	0.825
TT(s)	18.0 (17.4, 18.6)	18.0 (17.4, 18.6)	18.0 (17.4, 18.6)	0.647
D-dimer(mg/L)	0.27 (0.18, 0.47)	0.24 (0.16, 0.45)	0.30 (0.20, 0.49)	0.075
Fibrinogen(g/L)	2.85 (2.45, 3.48)	2.81 (2.39, 3.47)	2.95 (2.49, 3.55)	0.356
URBC	88 (30, 270)	91 (31.23, 264)	87 (30, 270)	0.853
IgA (g/L)	3.3 (2.6, 4.7)	3.2 (2.6, 4.4)	3.5 (2.6, 6.24)	0.422
IgG (g/L)	11.6 (9.4, 15.5)	12.1 (9.6, 16.3)	11.1 (9.3, 14.9)	0.261

IgM(g/L)	0.88 (0.59, 1.35)	0.89 (0.62, 1.42)	0.83 (0.57, 1.25)	0.117
C3 (g/L)	1.08 (0.94, 1.26)	1.10 (0.97, 1.23)	1.06 (0.92, 1.31)	0.944
C4 (g/L)	0.25 (0.20, 0.31)	0.25 (0.20, 0.30)	0.25 (0.20, 0.33)	0.741
RBP (mg/L)	48.0 (35.9, 61.5)	49.3 (37.9, 60.2)	46.3 (33.4, 63.1)	0.261
**Pathological parameter**				
Lee (1/2/3/4/5)	11/87/118/36/6	7/55/75/20/3	4/32/43/16/3	0.864
Hass (1/2/3/4/5)	34/111/73/31/9	19/74/46/16/5	15/37/27/15/4	0.515
M (0/1)	195/63	119/41	76/22	0.669
E (0/1)	255/3	158/2	97/1	1
S (0/1)	48/210	30/130	18/80	1
T (0/1/2)	219/29/10	135/19/6	84/10/4	0.955
C (0/1/2)	132/87/39	81/54/25	51/33/14	0.953
**Medications**				
RAAS inhibitor (%)	68 (26)	37 (23)	31 (32)	0.174
Steroids and/or immunosuppressants (%)	96 (37)	56 (35)	40 (41)	0.421

BMI Body mass index; SBP systolic blood pressure; DBP diastolic blood pressure; eGFR estimated glomerular filtration rate; BUN blood urea nitrogen; Scr serum creatine; 25(OH)D 25-hydroxy vitamin D; FBG fasting blood glucose; HbAc1 Glycosylated Hemoglobin; TG triglyceride; TC total cholesterol; LDL-C low-density lipoprotein cholesterol; HDL-C high-density lipoprotein cholesterol; WBC white blood cell; PT prothrombin time; INR International normalized ratio; APTT activated partial thromboplastin time; TT thrombin time; URBC urinary red blood cell; IgA Immunoglobulin A; IgG Immunoglobulin G; C3 complement 3; C4 complement 4; RBP Retinol binding protein; RAAS Renin-Angiotensin-Aldosterone System; MAKE Major adverse kidney events; D-Scr doubling of serum creatinine level;

Data were presented as the mean±standard, the median with interquartile range or counts and percentages. A two-tailed p<0.05 was considered statistically significant.

Supplementary Table 5. Baseline clinicopathological characteristics stratified by different TWA 25(OH) D level in validation set.

**Parameter**	**Total (n=172)**	**Low-TWA 25(OH)D group (＞44.8nmol/L，n=111)**	**High-TWA 25(OH)D group (＜44.8nmol/L，n=61)**	**P value**
Age (years)	40 (28, 49)	41 (31, 49)	33 (26, 46)	0.006
Gender (male/female)	79/93	52/59	27/34	0.869

MAKE, No. (%)	15 (8.7)	1 (0.9)	14 (23)	<0.001
**Comorbid disease**				
CKD stage (1/2/3a/3b/4)	101/36/15/12/8	65/28/8/8/2	36/8/7/4/6	0.065
Hypertension (%)	52 (30)	33 (30)	19 (31)	0.984
Cardiovascular diseases (%)	1 (0.6)	0 (0)	1 (1.6)	0.355
Diabetic mellitus	7 (4.1)	4 (3.6)	3 (4.9)	0.7
**Clinical parameter**				
BMI (kg/m2)	23.7 (21.3,26.2)	23.7 (21.7, 26.6)	23.7 (19.8, 27.0)	0.336
SBP (mmHg)	128 (118, 137)	126 (118, 136)	129 (116, 138)	0.92
DBP (mmHg)	82.3 ± 12.6	82.1 ± 12.4	82.6 ± 13.0	0.788
**Laboratory parameter**				
Urinary protein (mg/d)	863 (292,2018)	796 (246, 1700)	1262 (443, 3226)	0.021
eGFR (ml/min/1.73 m^2)	96 (67, 112)	96 (69, 109)	96 (57, 118)	0.884
BUN (mmol/L)	5.4 (4.4, 6.7)	5.5 (4.4, 6.7)	5.3 (4.2, 6.9)	0.92
Scr (μmol/L)	79.6 (61.4,100.5)	80.4 (62, 96.7)	79.0 (61.1, 123.3)	0.531
Uric acid (μmol/L)	356 (296, 440)	356 (299, 439)	356 (286, 444)	0.931
Serum albumin (g/L)	38.8 (34.4, 42.4)	39.5 (35.8, 42.6)	35.9 (33.7, 39.9)	0.002
Serum 25(OH)D (nmol/L)	36.7 (24.5, 50.2)	44.3 (31.3, 57.7)	23.0 (15.8, 35.6)	< 0.001
TWA 25(OH)D (nmol/L)	51.8 ± 19.3	62.8 ± 13.8	31.8 ± 9.3	< 0.001
FBG (mmol/L)	4.7 (4.4, 5.1)	4.8 (4.4, 5.2)	4.6 (4.3, 5.0)	0.112
TG (mmol/L)	1.33 (0.89, 1.99)	1.34 (0.96, 1.94)	1.31 (0.78, 2.12)	0.636
TC (mmol/L)	4.61 (3.95, 5.46)	4.62 (4.06, 5.40)	4.57 (3.81, 5.90)	0.925
LDL-C (mmol/L)	3.05 (2.59, 3.71)	3.05 (2.62, 3.63)	3.09 (2.45, 3.96)	0.878
HDL-C (mmol/L)	1.16 (0.94, 1.34)	1.18 (0.91, 1.35)	1.13 (0.94, 1.29)	0.953
Hemoglobin(g/L)	131.1 ± 21.7	133.4 ± 19.9	126.9 ± 24.2	0.076

WBC(×10^9/L)	6.5 (5.3, 7.7)	6.3 (5.1, 7.6)	6.6 (5.7, 7.6)	0.46
Neutrophil(×10^9/L)	3.9 (3.1, 5.0)	3.9 (3.0, 5.0)	3.9 (3.1, 5.2)	0.555
Neutrophil (%)	62.0 ± 8.9	61.8 ± 8.8	62.4 ± 9.2	0.682
Lymphocyte(×10^9/L)	1.8 (1.5, 2.1)	1.8 (1.5, 2.1)	1.9 (1.5, 2.2)	0.554
Lymphocyte (%)	29.2 ± 7.8	29.2 ± 7.7	29.1 ± 8.1	0.952
Monocyte(×10^9/L)	0.39 (0.31, 0.48)	0.4 (0.31, 0.48)	0.39 (0.28, 0.46)	0.353
Monocyte (%)	5.95 (5.05, 7.10)	6.10 (5.25, 7.10)	5.40 (4.50, 7.00)	0.054
Platelet (×10^9/L)	216 (188, 273)	214 (181, 273)	217 (190, 270)	0.901
PT(s)	11.7 (11.4, 12.2)	11.7 (11.5, 12.1)	11.7 (11.3, 12.3)	0.971
INR	1.02 (0.99, 1.06)	1.02 (1, 1.05)	1.02 (0.98, 1.06)	0.807
APTT(s)	27.6 (25.6, 29.2)	27.5 (25.4, 29.1)	27.9 (26.5, 29.2)	0.221
TT(s)	18.2 (17.5, 18.8)	18.4 (17.6, 18.8)	17.9 (17.2, 18.9)	0.136
D-dimer(mg/L)	0.26 (0.15, 0.45)	0.26 (0.16, 0.44)	0.29 (0.15, 0.51)	0.438
Fibrinogen(g/L)	2.80 (2.34, 3.28)	2.66 (2.33, 3.21)	2.97 (2.36, 3.48)	0.167
URBC	93 (33, 332)	94 (34, 289)	92 (28, 362)	0.85
IgA (g/L)	3.3 (2.5, 4.8)	3.5 (2.7, 5.4)	2.9 (2.2, 4.3)	0.019
IgG (g/L)	11.8 (9.4, 15.1)	13.0 (10.2, 16.1)	10.3 (8.6, 12.1)	< 0.001
IgM(g/L)	0.98 (0.61, 1.42)	1.01 (0.68, 1.46)	0.95 (0.52, 1.22)	0.108
C3 (g/L)	1.06 (0.92, 1.29)	1.08 (0.96, 1.29)	1.03 (0.88, 1.29)	0.243
C4 (g/L)	0.25 (0.20, 0.31)	0.25 (0.20, 0.31)	0.26 (0.21, 0.30)	0.681
RBP (mg/L)	51.0(39.7, 63.6)	48.8 (39.7, 62.3)	54.0 (41.0, 64.0)	0.576
**Pathological parameter**				
Lee (1/2/3/4/5)	6/51/91/20/4	3/30/66/9/3	3/21/25/11/1	0.099
Hass (1/2/3/4/5)	16/81/53/18/4	9/59/33/7/3	7/22/20/11/1	0.071
M (0/1)	125/47	78/33	47/14	0.438
E (0/1)	167/5	107/4	60/1	0.657
S (0/1)	31/141	20/91	11/50	1
T (0/1/2)	149/19/4	97/10/4	52/9/0	0.203
C (0/1/2)	92/55/25	60/33/18	32/22/7	0.571
**Medications**				
RAAS inhibitor (%)	48 (28)	30 (27)	18 (30)	0.865
Steroids and/or immunosuppressants (%)	79 (46)	46 (41)	33 (54)	0.152

BMI Body mass index; SBP systolic blood pressure; DBP diastolic blood pressure; eGFR estimated glomerular filtration rate; BUN blood urea nitrogen; Scr serum creatine; 25(OH)D 25-hydroxy vitamin D; FBG fasting blood

glucose; HbAc1 Glycosylated Hemoglobin; TG triglyceride; TC total cholesterol; LDL-C low-density lipoprotein cholesterol; HDL-C high-density lipoprotein cholesterol; WBC white blood cell; PT prothrombin time; INR International normalized ratio; APTT activated partial thromboplastin time; TT thrombin time; URBC urinary red blood cell; IgA Immunoglobulin A; IgG Immunoglobulin G; C3 complement 3; C4 complement 4; RBP Retinol binding protein; RAAS Renin-Angiotensin-Aldosterone System; MAKE Major adverse kidney events; D-Scr doubling of serum creatinine level;

Data were presented as the mean±standard, the median with interquartile range or counts and percentages. A two-tailed $p<0.05$ was considered statistically significant.

Supplementary Table 6. Basic information of 25(OH)D and its active form 1,25(OH)D

Molecular Name	Molecular Formula	Molecular Weight	2D Structure	3D Structure
25(OH)D(3)	C27H44O2	400.6 g/mol	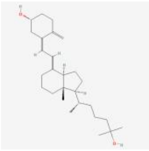	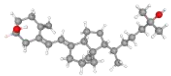
1,25(OH)D(3)	C27H44O3	416.6 g/mol	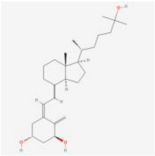	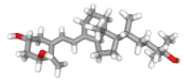

25(OH)D(3) , 25-hydroxy vitamin D3; 1,25(OH)D(3), 1,25 dihydroxy vitamin D3

Supplementary Table 7. Expression of target genes in different cell types.

	PTC		PODO		SMC		PC	
	Log_2FC	P-adjust	Log_2FC	P-adjust	Log_2FC	P-adjust	Log_2FC	P-adjust
AGTR1	-0.242	1	0.000	1	0.900	1	0.174	1
CCR2	0.238	1	0.000	1	0.000	1	0.000	1
CSNK2B	0.094	1	-2.031	1	-0.575	1	-0.157	1
FCGRT	-0.057	1	-0.111	1	5.780	1	-0.223	1
NFKB1	1.079	<0.001	-1.998	1	4.420	1	0.252	1
NR4A1	0.856	<0.001	1.729	1	6.149	1	-0.087	1
PDGFRB	0.459	1	0.725	1	4.968	1	0.493	1
PIK3CG	0.170	1	0.000	1	0.000	1	-0.084	1
PRKCD	0.042	1	-2.960	1	-3.075	1	-0.074	1
PTGER2	0.531	1	0.000	1	1.789	1	0.460	1
RASGRP1	1.682	1	3.122	1	2.984	1	-0.083	1
SERPINE1	1.401	1	0.000	1	2.376	1	0.380	1
	MON		MES		LOH		IC	
	Log_2FC	P-adjust	Log_2FC	P-adjust	Log_2FC	P-adjust	Log_2FC	P-adjust
AGTR1	0.000	1	0.000	1	-0.907	1	0.000	1

CCR2	0.808	1	0.000	1	0.629	1	0.000	1
CSNK2B	1.095	1	-0.821	1	-0.187	1	-0.545	1
FCGRT	-0.927	1	-0.655	1	-0.774	1	0.251	1
NFKB1	5.555	1	0.905	1	-0.193	1	1.086	1
NR4A1	2.701	1	0.029	1	0.937	1	0.618	1
PDGFRB	0.000	1	0.442	1	0.000	1	0.000	1
PIK3CG	0.000	1	0.000	1	0.280	1	0.000	1
PRKCD	-1.041	1	2.059	1	-1.256	1	0.883	1
PTGER2	3.222	1	0.320	1	0.429	1	0.000	1
RASGRP1	3.937	1	-1.734	1	-0.276	1	-1.283	1
SERPINE1	0.872	1	2.926	1	0.000	1	0.000	1
	MC		EC		DTC			
	Log$_2$FC	P-adjust	Log$_2$FC	P-adjust	Log$_2$FC	P-adjust		
AGTR1	0.000	1	-3.056	1	0.000	1		
CCR2	4.429	1	0.786	1	0.000	1		
CSNK2B	-0.140	1	-0.361	1	0.458	1		
FCGRT	0.246	1	-0.897	1	-0.425	1		
NFKB1	-0.589	1	1.019	1	-0.295	1		
NR4A1	-0.224	1	1.362	1	1.830	1		
PDGFRB	0.000	1	-1.840	1	2.115	1		
PIK3CG	0.568	1	1.384	1	0.000	1		
PRKCD	-0.286	1	-2.771	1	-1.797	1		
PTGER2	-3.741	<0.001	-0.824	1	0.000	1		
RASGRP1	-1.297	1	-0.239	1	2.265	1		
SERPINE1	-3.387	1	0.880	1	0.000	1		

PTC, proximal tubule cells; LOH, loop of Henle cells; PC, principal cells; IC, intercalated cells; DTC, distal tubule cells; EC, endothelial cells; PODO, podocytes; MES, mesangial cell; SMC , smooth muscle cells; MC, macrophages; MON, monocytes; AGTR1, angiotensin II receptor type 1; CCR2, c-c motif chemokine receptor 2; CSNK2B, casein kinase 2 beta; FCGRT, fc gamma receptor and transporter; NFKB1, nuclear factor kappa b subunit 1; NR4A1, nuclear receptor subfamily 4 group a member 1; PDGFRB, platelet derived growth factor receptor beta; PIK3CG, phosphatidylinositol-4,5-bisphosphate 3-kinase catalytic subunit gamma; PRKCD, protein kinase c delta; PTGER2, prostaglandin e receptor 2; RASGRP1, RAS guanyl releasing protein 1; SERPINE1, serpin family e member 1

Supplementary Table 8. Affinity of gene binding to 25(OH)D and the active form 1,25(OH)D.

**The Targets**	**Affinity (kcal/mol)**		**The Targets**	**Affinity (kcal/mol)**	
	**to 25(OH)D**	**to 1,25(OH)D**		**to 25(OH)D**	**to 1,25(OH)D**
FCGRT	-7.4	-5.7	AGTR1	-5.8	-5.4
CYP19A1	-7.2	-6.0	CSNK2B	-5.7	-6.4
CXCR3	-6.6	-4.9	RASGRP1	-5.4	-4.6
PIK3CG	-6.5	-6.1	CCR2	-6.0	-4.8
PDGFRB	-6.1	-5.5	SERPINE1	-5.0	-6.1
PRKCD	-6.0	-5.7			

25(OH)D , 25-hydroxy vitamin D; 1,25(OH)D, 1,25 dihydroxy vitamin D; AGTR1, angiotensin II receptor type 1;

CCR2, c-c motif chemokine receptor 2; CSNK2B, casein kinase 2 beta; FCGRT, fc gamma receptor and transporter; NFKB1, nuclear factor kappa b subunit 1; NR4A1, nuclear receptor subfamily 4 group a member 1; PDGFRB, platelet derived growth factor receptor beta; PIK3CG, phosphatidylinositol-4,5-bisphosphate 3-kinase catalytic subunit gamma; PRKCD, protein kinase c delta; PTGER2, prostaglandin e receptor 2; RASGRP1, RAS guanyl releasing protein 1; SERPINE1, serpin family e member 1.
